# Maternal pre-pregnancy body mass index and risk of preterm birth: a collaboration using large routine health datasets

**DOI:** 10.1186/s12916-023-03230-w

**Published:** 2024-01-05

**Authors:** R. P. Cornish, M. C. Magnus, S. K. Urhoj, G. Santorelli, L. G. Smithers, D. Odd, A. Fraser, S. E. Håberg, A. M. Nybo Andersen, K. Birnie, J. W. Lynch, K. Tilling, D. A. Lawlor

**Affiliations:** 1 Population Health Sciences, Bristol Medical School, University of Bristol, Oakfield House, Oakfield Road, Bristol, BS8 2BN UK; 2 MRC Integrative Epidemiology Unit, University of Bristol, Bristol, UK; 3 Centre for Fertility and Health, Norwegian Institute of Public Health, Oslo, Norway; 4 Department of Public Health, Faculty of Health Sciences, University of Copenhagen, Copenhagen, Denmark; 5 Bradford Institute for Health Research, Bradford Royal Infirmary, Bradford, UK; 6 School of Public Health, University of Adelaide, Adelaide, Australia; 7 School of Health and Society, University of Wollongong, Wollongong, Australia; 8 Division of Population Medicine, Cardiff University School of Medicine, Cardiff, UK

**Keywords:** Body mass index, Maternal, Pre-pregnancy, Preterm birth, Non-linear, Parity

## Abstract

**Background:**

Preterm birth (PTB) is a leading cause of child morbidity and mortality. Evidence suggests an increased risk with both maternal underweight and obesity, with some studies suggesting underweight might be a greater factor in spontaneous PTB (SPTB) and that the relationship might vary by parity. Previous studies have largely explored established body mass index (BMI) categories. Our aim was to compare associations of maternal pre-pregnancy BMI with any PTB, SPTB and medically indicated PTB (MPTB) among nulliparous and parous women across populations with differing characteristics, and to identify the optimal BMI with lowest risk for these outcomes.

**Methods:**

We used three UK datasets, two USA datasets and one each from South Australia, Norway and Denmark, together including just under 29 million pregnancies resulting in a live birth or stillbirth after 24 completed weeks gestation. Fractional polynomial multivariable logistic regression was used to examine the relationship of maternal BMI with any PTB, SPTB and MPTB, among nulliparous and parous women separately. The results were combined using a random effects meta-analysis. The estimated BMI at which risk was lowest was calculated via differentiation and a 95% confidence interval (CI) obtained using bootstrapping.

**Results:**

We found non-linear associations between BMI and all three outcomes, across all datasets. The adjusted risk of any PTB and MPTB was elevated at both low and high BMIs, whereas the risk of SPTB was increased at lower levels of BMI but remained low or increased only slightly with higher BMI. In the meta-analysed data, the lowest risk of any PTB was at a BMI of 22.5 kg/m^2^ (95% CI 21.5, 23.5) among nulliparous women and 25.9 kg/m^2^ (95% CI 24.1, 31.7) among multiparous women, with values of 20.4 kg/m^2^ (20.0, 21.1) and 22.2 kg/m^2^ (21.1, 24.3), respectively, for MPTB; for SPTB, the risk remained roughly largely constant above a BMI of around 25–30 kg/m^2^ regardless of parity.

**Conclusions:**

Consistency of findings across different populations, despite differences between them in terms of the time period covered, the BMI distribution, missing data and control for key confounders, suggests that severe under- and overweight may play a role in PTB risk.

**Supplementary Information:**

The online version contains supplementary material available at 10.1186/s12916-023-03230-w.

## Background

Preterm birth (PTB; birth before 37 completed weeks gestation) affects around 10% of pregnancies worldwide. It is the leading cause of perinatal mortality and morbidity, and of childhood death up to 5 years [1, 2]. Recent increases in PTB [2, 3] may be, in part, related to the obesity epidemic, with other possible factors including increased maternal age at pregnancy and changes in obstetric practice resulting in increased rates of preterm caesarean delivery [4, 5]

PTB can be medically indicated (MPTB) or spontaneous (SPTB). MPTB is driven by obstetric interventions (induction of labour or planned caesarean section) related to pregnancy complications such as pre-eclampsia or gestational diabetes and thus may be higher in women with overweight or obesity [6]. Known risk factors for SPTB include infection and inflammation, genetic factors, and some lifestyle factors such as stress, smoking and alcohol intake, although the cause is often unknown [4, 5]. While the detrimental effects of MPTB are a trade off with detrimental effects of continued pregnancy in the presence of such conditions, SPTB is a major concern obstetrically because of its unpredictable nature.

Evidence from systematic reviews suggests an increased risk of PTB with both maternal overweight/obesity and underweight [7–12], with some studies indicating that underweight might be a greater factor than obesity in SPTB [7–9, 13–16]. In addition, there is evidence that the association of BMI with PTB may vary by parity, with some suggestion of a stronger association of obesity with SPTB among nulliparous women [17, 18] and of different associations of underweight with SPTB and MPTB among nulliparous and parous women [19], although the numbers of women with underweight in these studies have been small. Previous studies have largely explored established BMI categories and not attempted to identify the BMI with lowest risk or compared associations across countries with different levels of obesity. Our aim was to compare associations of maternal BMI with PTB, SPTB and MPTB, among pregnancies in nulliparous and parous women separately, across populations with differing characteristics, and to identify the optimal BMI with lowest risk for these outcomes.

## Methods

### Datasets

**Fig. 1:**
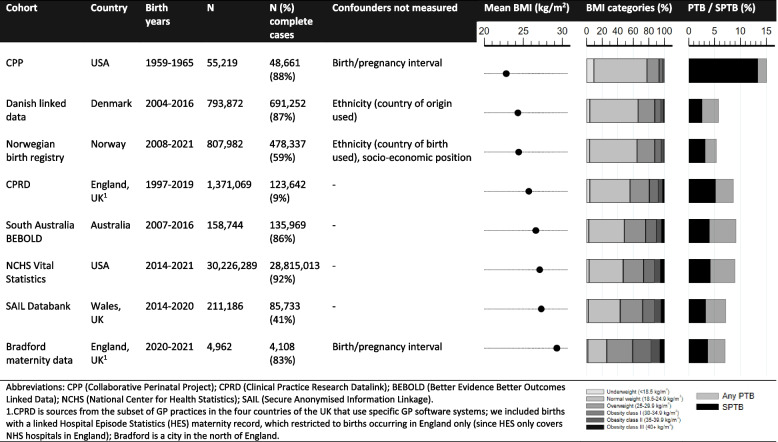
Key characteristics of the datasets

The datasets are described in Fig. 1, summarising differences in key characteristics such as: years covered; BMI distribution; availability of confounders; and data completeness, with further details regarding missing data given in Additional file 1: Supplementary Figure S1. Datasets ranged in size from just under 5000 to over 27 million pregnancies and included three UK, two US and one dataset each from Australia, Norway and Denmark. The datasets from Norway and Denmark and one from the USA (US vital statistics data) included all registered births across the countries during the study period. The years covered varied, with all except one (Collaborative Perinatal Project, USA, 1959–1965) including recent data.

### Collaborative Perinatal Project (CPP)

CPP recruited just over 46,000 women with 59,391 pregnancies from twelve US centres providing prenatal care between 1959 and 1965 [20, 21]. Gestational age was based on last menstrual period (LMP) and was recorded in number of weeks, rounded to the nearest week. BMI was calculated from height (measured) and weight (self-reported) recorded at study enrolment, which was at the first antenatal visit for the majority of pregnancies.

### Danish linked data

We included all live births and stillbirths in Denmark between 2004 and 2016, using linked information from the Danish Medical Birth Registry [22] (MBR) and population registers held by Statistics Denmark. In the MBR, gestational age is based on routine ultrasound measures at 18–20 weeks gestation or LMP for the small proportion of pregnancies with no ultrasound measurements. Maternal pre-pregnant BMI was calculated from self-reported height and weight recorded at the first antenatal appointment.

### Norwegian birth registry

We used data between 2008 and 2021 from the Birth Registry of Norway (MBRN), which includes mandatory registrations of pregnancies in Norway ending after 12 completed gestational weeks [23]. Gestational age at birth was based on routine ultrasound measures at 18 weeks gestation, or LMP for those without ultrasound-based estimates (< 5% of pregnancies). BMI was calculated from maternal self-reported pre-pregnancy height and weight recorded in antenatal care records at 8–12 weeks gestation.

### Clinical Practice Research Datalink (CPRD)

CPRD is a population-based database of primary care data from across the UK [24] linked to other datasets. We included all pregnancies from the CPRD (GOLD) Pregnancy Register [25] for the period 1997–2019 resulting in a live or still birth and with a linked record in the Hospital Episode Statistics (HES) maternity data; the latter covers NHS hospitals in England only. Gestational age was based on routine ultrasound measures taken between 10 and 14 weeks’ gestation or LMP for the minority with no ultrasound measurements. BMI was obtained from weight and height measurements recorded in the primary care data. We required these to be from a maximum of 12 months pre-pregnancy up to a maximum of 15 weeks gestation and, where recorded more than once during this period, took measurements from closest to the time of conception.

### South Australian Better Evidence Better Outcomes Linked Data (BEBOLD) platform

Pregnancy data was obtained from the BEBOLD platform, which includes the South Australian Perinatal Statistics Collection 2007–2016, a mandatory collection of all births at least 400 g or 20 weeks gestation [26]. Gestational age was determined from LMP if dates were deemed reliable and early ultrasound (up to 20 weeks) otherwise. BMI was calculated from weight and height measured and recorded at the first antenatal visit, attended prior to 14 weeks gestation in approximately 85% of pregnancies.

### US National Center for Health Statistics Vital Statistics (NCHS) data

We used publicly available birth and foetal death datasets from 2014 to 2021. These include information from mandatory registrations of all births and foetal deaths; for most states this includes foetal deaths of at least 350 g and/or 20 weeks gestation [27]. Gestational age was based on routine ultrasound measurements or last menstrual period for the small proportion (< 1%) with no ultrasound data. Maternal pre-pregnancy weight and height were self-reported by the women at the time of birth (see Additional file 1 for further explanation of this).

### Secure Anonymised Information Linkage (SAIL) Databank

The SAIL databank contains de-identified health and administrative data on the population of Wales, UK [28, 29]. We included pregnancies resulting in a live or still birth from 2014 to 2020 with a birth record in either the Maternity Indicators Dataset (MID) [30] (data from the first antenatal assessment at 8–12 weeks pregnancy plus labour and birth) or the National Community Child Health (NCCH) database (birth registration and other data). Gestational age was based on routine ultrasound measurements taken at 10–14 weeks gestation or LMP for the minority of pregnancies where no ultrasound measures were available. BMI was obtained from weight and height measurements recorded in the MID (from the first antenatal visit) or from the primary care data; if from the latter, we required these to be from a maximum of 12 months pre-pregnancy up to a maximum of 15 weeks gestation and, where recorded more than once during this period, took measurements from closest to the time of conception.

### Bradford maternity data

Maternity record data for all births at Bradford Royal Infirmary (BRI) between January 2020 and March 2021 were obtained from BRI Informatics Department. Gestational age was based on routine ultrasound measurements taken at 10–14 weeks gestation or LMP for the minority of pregnancies where no ultrasound measures were available. BMI was derived from height and weight measured at the first antenatal appointment attended from 8 to 12 weeks gestation.

Further details of each dataset are provided in the supplementary materials (Additional file 1: Supplementary text A.1).

### Outcomes

The primary outcome measures were any PTB, SPTB (delivery < 37 completed weeks, with spontaneous onset of labour) and MPTB (labour induced or delivery initiated by caesarean section prior to onset of labour). Foetal deaths occurring up to 23 weeks, 6 days of gestation were excluded since these were absent or incomplete in most datasets. Secondary outcomes were very PTB, SPTB and MPTB (< 32 weeks).

### Exposure

The exposure was maternal pre- or early pregnancy BMI, calculated from self-reported or measured pre-pregnancy or early pregnancy weight and height (details in Supplementary materials).

### Covariates

The following confounders were identified a priori [31] based on the definition that they are known or plausible causes of variation early/pre-pregnancy BMI and PTB: maternal age at birth, ethnicity, smoking, socio-economic position (SEP), and, among pregnancies in parous women, parity and birth or birth interval. The availability of these confounders varied, as summarised in Fig. 1 and with further details in supplementary materials (Additional file 1). Because of its strong association with PTB, pregnancy size (singleton/multiple) was included as a covariate to increase precision. We decided a priori to maximise confounder adjustment within each dataset by not harmonising variables across datasets (where recorded differently or unavailable) but using the most detailed measures within each.

### Statistical methods

All analyses were carried out with pregnancy as the unit of analysis and were carried out separately in nulliparous and parous women. For the primary analysis, multivariable logistic regression using fractional polynomials [32] with up to three powers of BMI was used to examine the association between BMI and any PTB, SPTB and MPTB. Within each dataset we adjusted for all available confounders. With the exception of the NCHS Vital Statistics data, where there was no (person-level) ID variable, robust standard errors were used to take account of the fact that some women had more than one recorded pregnancy per dataset. For each outcome, we chose an optimal model (in terms of the fractional polynomial; all models included all available confounders) that fit well in all datasets (and was potentially the best fitting model in several). Once the optimal model for each outcome had been selected, we carried out a multivariate, random effects meta-analysis with inverse variance weighting on the aggregate data. It was not possible to combine individual-level data, as most datasets had to be analysed on secure servers in different locations. Confounder-adjusted risks of any PTB, MPTB and SPTB were calculated from the optimal model and plotted against BMI. Where possible, the same reference group was used and consisted of singleton pregnancies, maternal age 25–29 years, non-smoker, pregnancy/birth interval not < 12 months, White/Caucasian and, among parous women, parity equal to one. For SEP, which was measured in various ways, the reference category was the median group. In the Danish and Norwegian datasets, where country of origin was measured, but not ethnicity, originating from Denmark/Norway was the reference group. Where feasible, the estimated BMI at which the risk of each outcome was lowest was calculated via differentiation and a 95% confidence interval (CI) obtained using bootstrapping (details in Additional file 1: Supplementary text A.2).

We conducted two secondary analyses. Firstly, we used standard WHO BMI categories (underweight < 18.5 kg/m^2^, healthy weight 18.5–24.9, overweight 25–29.9, obesity class I 30–34.9, obesity class II 35–39.9, obesity class III 40 +), to enable our results to be compared to other publications. Secondly, we examined very PTB (< 32 completed weeks gestation), as this is related to more adverse outcomes than births from 33 to < 37 weeks [33].

Various sensitivity analyses were conducted. Firstly, to account for the fact that a woman with a MPTB could not have a SPTB and vice versa, models for SPTB were weighted by the inverse of one minus the probability of being a MPTB; conversely, models for MPTB were weighted by the inverse of one minus the probability of being a SPTB. The models for the weights included the same variables as the analysis model. Secondly, we carried out analyses excluding (i) stillbirths, (ii) post term deliveries (≥ 42 completed weeks gestation) and (iii) multiple births. Finally, because the CPP was carried out in the 1960s, with all other datasets contributing recent data, reflecting more contemporary practice and monitoring, we repeated the meta-analyses excluding this dataset.

We hypothesised that, within datasets, some covariates—particularly BMI, ethnicity, smoking—might be missing not at random (specifically, less likely to be missing if individuals were not from an ethnic minority group, were a non-smoker or, depending on the source of BMI, more or less likely to be missing if individuals had either a very low or high BMI). Thus, we decided a priori to use a complete case analysis in all datasets because in this situation (covariates missing not at random), multiple imputation would produce bias, whereas a complete case logistic regression gives unbiased estimates unless the chance of being a complete case depends jointly on the exposure and outcome [34, 35] (i.e. in our case unless the relationship between BMI and the likelihood of it being missing were influenced by whether or not an individual subsequently had a preterm birth outcome), which we thought unlikely.

All analyses were carried out in Stata; meta-analysis used Stata’s mvmeta command [36].

## Results

Between 68% (CPRD) and 100% (South Australian BEBOLD) of pregnancies had gestational age at delivery recorded. In most datasets, BMI had the most missing data and between 9% (CPRD) and 92% (US Vital Statistics) were complete cases (Fig. 1; Additional file 1: Supplementary Figure S1). In CPRD, because BMI came from primary care data, thus relying on weight to have been measured near the time of conception as part of routine care, this information was only available for a small proportion of pregnancies. Additional file 1: Supplementary Tables S1 to S8 give characteristics of the whole sample and complete cases for each dataset; across all datasets, characteristics were similar.

The overall risks of PTB and SPTB among complete cases were lowest in the Norwegian birth registry and Danish linked data, respectively (5.4% PTB Norway, 2.6% SPTB Denmark), and highest in the Collaborative Perinatal Project (CPP) (15.2% and 13.4%, respectively) (Fig. 1). The risk of MPTB ranged from 1.6% (CPP) to 5.1% (US Vital Statistics). In CPP, SPTB accounted for around 90% of PTB; in the other datasets, it ranged from 43 to 58%. The mean BMI ranged from 22.8 kg/m^2^ (CPP) to 29.3 kg/m^2^ (Bradford) (Fig. 1; Additional file 1: Supplementary Tables S1-S8).

### Main results

**Fig. 2:**
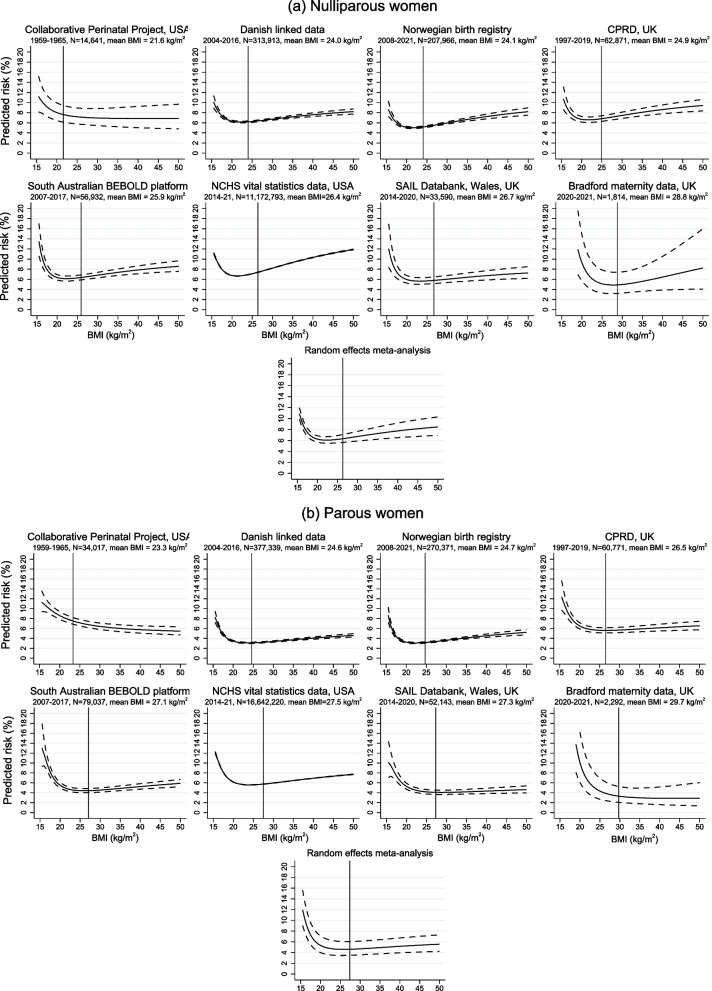
Association of pre-pregnancy BMI with risk of any preterm birth among (**a**) nulliparous and (**b**) parous women

**Fig. 3:**
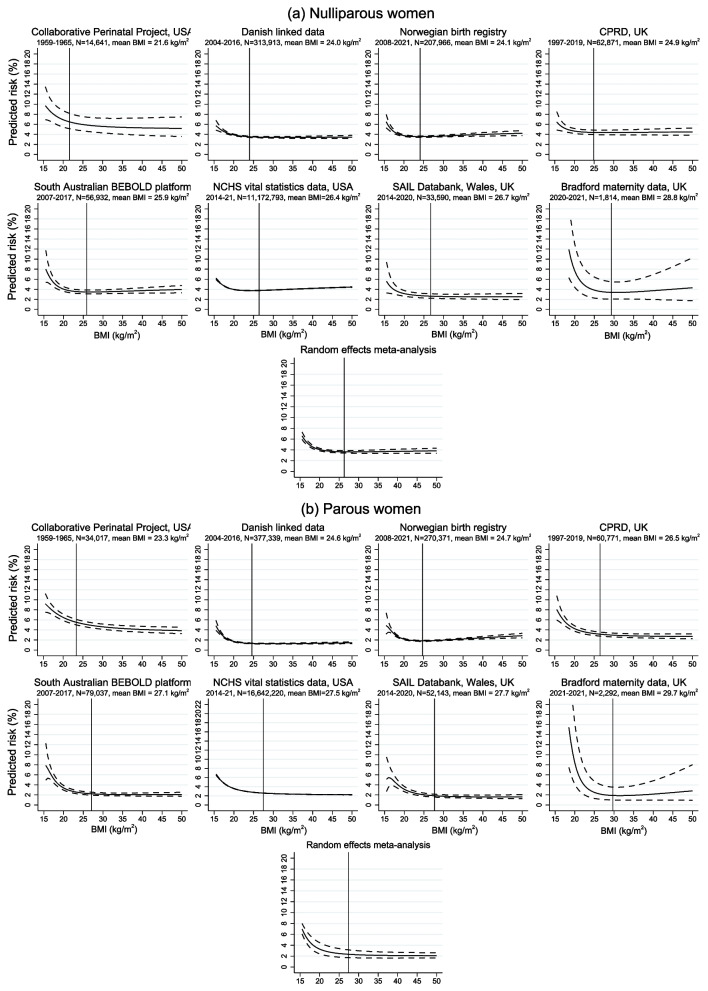
Association of pre-pregnancy BMI with risk of spontaneous preterm birth among (**a**) nulliparous and (**b**) parous women

**Fig. 4:**
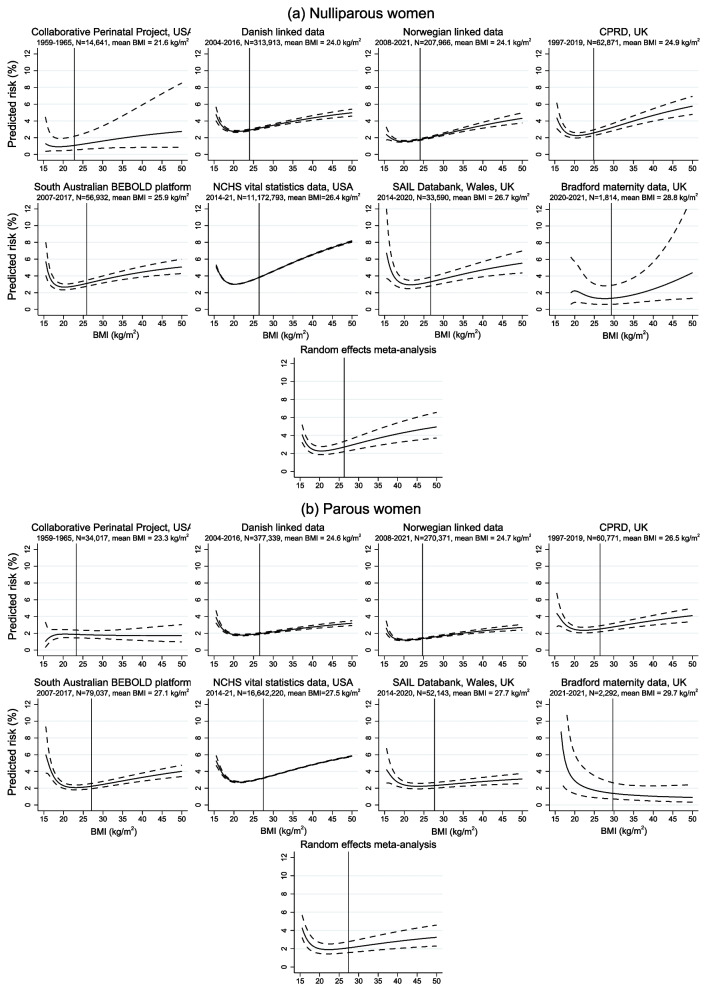
Association of pre-pregnancy BMI with risk of medically indicated preterm birth among (**a**) nulliparous and (**b**) parous women

Figures 2, 3 and 4 show the association of BMI with risk of any PTB, SPTB and MPTB among nulliparous and parous women in each dataset obtained from the optimal fractional polynomial model. Full details of the fractional polynomial models and how we reached the final model are provided in Supplementary Text A.2 and Supplementary Tables S11 and S12, with results given in Additional file 1: Supplementary Tables S13 and S14. On each graph, the mean BMI (among nulliparas and paras, as applicable) in that dataset is plotted as a reference line. Across all datasets except CPP, the risk of any PTB increased with lower and higher BMI, with the latter largely driven by an increase in MPTB with increasing BMI from the lowest risk levels. In contrast, the risk of SPTB was higher at lower BMIs but remained low or increased only slightly with higher BMI. The overall pattern was similar among nulliparous and multiparous women, although the increased risk at low BMI was, in general, more marked among parous women and, in contrast, the increase with higher BMI was slightly more marked among nulliparas. The meta-analysed curves are also shown on these figures. There was relatively high heterogeneity in the estimates, likely due to variability in the prevalence of PTB, the exact nature of the curves in terms of the location of the minimum point (if any) and the extent to which the risk increased at low and/or high BMIs, and the fact that the estimates were very precise in some datasets. Despite this heterogeneity, there was generally relative consistency in the overall pattern of risk with BMI.

Table 1 shows the BMI, with 95% CI, at which the predicted risk for any PTB and MPTB was lowest. It was not possible to calculate this for SPTB in most datasets or in the meta-analysed results because the risk did not vary across most of the BMI distribution. The lowest predicted risk of any PTB, where calculable, was at a BMI between 21.2 and 27.8 kg/m^2^ among nulliparous women, between 22.3 and 28.7 kg/m^2^ among parous women, and in the meta-analysed data, it was at 22.5 and 25.9 kg/m^2^ (for nulliparous and parous women, respectively). The lowest risk of MPTB, where calculable, occurred at BMIs between 18.8 and 27.6 kg/m^2^ in nulliparous women, between 20.2 and 23.5 kg/m^2^ in parous women, and was at a BMI of 20.4 and 22.2 kg/m^2^ (respectively) in the meta-analysed data.

**Table 1:** Body mass index (BMI) (95% CI) at which the predicted risk of outcomes was lowest among pregnancies in nulliparous and parous women

Dataset	Mean BMI (kg/m^2^)	Any PTB	MPTB
Nulliparous			
CPP	21.6	NA^a^	18.8 (^b^)
Danish linked data	24.0	22.3 (21.7, 23.6)	20.7 (20.5, 21.1)
Norwegian birth registry	24.1	21.4 (20.7, 23.1)	20.0 (19.6, 20.9)
CPRD	24.9	21.6 (20.9, 23.1)	20.6 (20.3, 21.4)
South Australian BEBOLD	25.9	22.3 (21.1, 26.7)	20.3 (20.0, 20.9)
NCHS data (USA)	26.4	21.2 (21.2, 21.3)	20.2 (20.2, 20.3)
SAIL databank	26.7	23.2 (21.6, 28.5)	21.6 (20.1, 42.5)
Bradford	28.8	27.8 (20.1, ^b^)	27.6 (^b^)
**Meta-analysed data**		**22.2 (21.5, 23.5)**	**20.4 (20.0, 21.1)**
Parous women			
CPP	23.3	NA^a^	NA^c^
Danish linked data	24.6	23.8 (22.9, 25.2)	21.9 (21.4, 22.8)
Norwegian birth registry	24.7	22.3 (21.4, 24.2)	20.2 (20.0, 20.5)
CPRD	26.5	26.5 (23.3, 41.8)	22.4 (20.7, 30.1)
South Australian BEBOLD	27.1	26.0 (22.8, 38.3)	23.1 (21.3, 28.3)
NCHS data (USA)	27.5	24.1 (23.9, 24.2)	21.5 (21.2, 21.8)
SAIL databank	27.7	28.7 (23.9, ^b^)	23.5 (21.1, ^b^)
Bradford	29.7	NA^a^	NA^a^
**Meta-analysed data**		**25.9 (24.1, 31.7)**	**22.2 (21.1, 24.3)**

^a^Risk decreased across the whole BMI range

^b^Limit(s) outside the observed BMI range due to high variability in the fractional polynomial terms

^c^Insufficient data

### Secondary analyses

The risks of PTB, SPTB and MPTB and adjusted odds ratios using BMI groups are given in Additional file 1: Supplementary Tables S9-S10 and S15-S16, respectively. The patterns reflect those shown in Figs. 2, 3 and 4, with an increased risk, relative to normal weight, of all three outcomes with underweight and an increasing risk of any PTB and MPTB, but not SPTB, with increasing BMI. The adjusted odds ratios for very PTB, SPTB and MPTB are given in Additional file 1: Supplementary Tables S17 and S18. For parous women, these show similar non-linear patterns to those seen for < 37 completed weeks. In contrast, for nulliparous women, the increased risk with underweight, particularly for SPTB, appeared to be weaker (in most datasets) when considering < 32 weeks than < 37 weeks whereas the increased risk of any PTB and MPTB with higher BMI was slightly stronger (and was also present for SPTB in several datasets) for < 32 weeks compared to < 37 weeks.

### Sensitivity analyses

The results from the sensitivity analyses were similar to the overall results (Additional file 1: Supplementary Tables S19-S26). Excluding CPP from the meta-analysis made the slope of the curve for any PTB slightly steeper at higher BMIs and reduced the estimated risk at very low BMIs among parous women but had no noticeable impact for nulliparas or for SPTB and MTPB (Additional file 1: Supplementary Figures S2 and S3).

## Discussion

We have examined the relationship between maternal pre-pregnant BMI and any PTB, SPTB and MPTB in several large datasets from different countries, with different sources of potential bias, and have shown non-linear associations with all three outcomes, across all datasets. The higher risk of any PTB at higher BMI was driven by MPTB, whereas the risk of SPTB was increased at lower levels of BMI but remained low or increased only slightly with higher BMI. Overall, the increased risk at low BMI was slightly more marked among parous women, whereas the increased risk (of any PTB and MPTB) with higher BMI was more marked among nulliparas. The key exception to the pattern for any PTB was in the CPP, where a large majority of the preterm births were spontaneous—so the relationship of BMI with any PTB followed that for SPTB, with an increased risk only among women with underweight. CPP was based on births between 1959 and 1965, which is around the time that gestational diabetes was first being described and acknowledged [37]. Similarly, the routine measuring of blood pressure and proteinuria antenatally was not common until the 1960s [38]. Hence, MPTB would be expected to be low in this dataset.

In the meta-analysed data, the lowest predicted risks of any PTB were at BMIs of 22.5 and 25.9 kg/m^2^ for nulliparous and parous women, respectively; for MTPB, they were at BMIs of 20.4 and 22.2 kg/m^2^. For SPTB, the risk remained relatively constant or increased only slightly for BMIs above 25–30 kg/m^2^ in both nulliparous and parous women. Taken together, these suggest that a healthy BMI to prevent either MPTB or SPTB would be between 20 and 30 kg/m^2^. The patterns of association were consistent across the datasets, despite the fact that they reflect different stages of the obesity epidemic, as indicated by the prevalence of overweight and obesity, and had different potential sources of bias due to varying proportions of missing data, measurement error in gestational age and BMI, and possible residual confounding.

Our findings are broadly consistent with previous studies that have explored associations of underweight, overweight or obesity using conventional BMI categories, showing an increased risk of PTB among women with underweight as well as with obesity [6–16], with the increased risk with obesity being largely a result of medically indicated PTB [6, 7]. To our knowledge, two studies have examined the relationship using BMI as a continuous variable. One used locally weighted scatterplot smoothing to examine the association with PTB and found that the minimum risk occurred at a BMI of ~ 23.5 kg/m^2^ [39]. The other applied restricted cubic splines to the US vital statistics data, including births between 2016 and 2018, and found the risk of PTB increased with both low and high BMIs, and was lowest at a BMI of ~ 24 kg/m^2^ [40]. Neither explored associations separately for SPTB and MPTB or by parity.

We found some suggestion for a differing relationship between BMI and very PTB (< 32 weeks) compared to < 37 weeks, particularly among nulliparous women, although these results were not consistent across all datasets. Possible reasons for this could be that risk factors for very PTB may be different or differentially important (for example, severe pre-eclampsia, placental pathology, maternal morbidity or severe conditions in the foetus).

Pre-pregnancy overweight and obesity are both associated with an increased risk of gestational hypertension and gestational diabetes [12, 40], which are associated with increased risk of induction of labour and/or planned caesarean section. This likely explains (some of) the increased risk of MPTB with higher BMI. Women with underweight can have difficulty conceiving and, when they do, are at greater risk of foetal growth restriction and PTB. This may be because of underlying maternal chronic diseases complicating the pregnancy [41] or maternal undernutrition resulting in impaired foetal growth [42]; these mechanisms likely explain the observed association of lower, but not higher, BMI with SPTB. Overall patterns were similar in pregnancies to women who were nulliparous and those who were parous, with pre-/early-pregnancy BMI between 20 and 30 kg/m^2^ minimising PTB risk.

The strengths of this work include the inclusion of large datasets (with differing sources and extent of potential bias) from different countries with varying prevalence of obesity. We have used BMI as a continuum to examine non-linear associations and have been able to explore associations with any, MPTB and SPTB. The datasets were generally derived from routine health data, thus minimising the risk of selection bias. That said, selection bias could have arisen due to missing data in some datasets. We undertook complete case analyses because, as stated above, we considered this the least biased approach due to confounders, and potentially BMI (in some datasets), possibly being missing not at random, but acknowledge that there was large variation in the extent of missing data, particularly for BMI. However, the similarity of non-linear associations across the datasets, despite these large variations in the extent of missing data suggests that any resulting bias is unlikely to have had a major impact on the general pattern of our findings. As such, the results should be generalisable to the populations from which they are drawn. In CPP and the Australian data, gestational age was estimated (or predominantly estimated for the latter) using LMP, which has been found to be less accurate, in general, than using early ultrasound measures [43]; further, in CPP gestational age was rounded to the nearest week (not completed weeks), which would misclassify some preterm births. On the other hand, ultrasound dating has been shown to be less accurate in women with obesity, tending to underestimate rates of preterm birth [44], which would mean the risk relative to normal weight might be underestimated for these women. In some datasets, weight and height were self-reported, which may be subject to misreporting (measurement error). Although agreement between reported and measured weight tends to be high [45], women with overweight/obesity are more likely to underreport their weight and women with underweight to overreport their weight [46]. This would tend to result in the risk being overestimated at both low and high BMIs. In addition, in the CPRD data and in just under a quarter of the pregnancies in the SAIL data, weight could have been measured up to 12 months prior to conception or up to 15 weeks gestation, which would also contribute to measurement error in BMI due to changes in BMI between the time of measurement and the time of conception. To maximise confounder adjustment in each dataset, we did not harmonise these to the lowest common denominator. However, residual confounding is possible as some measures were missing or had limited detail in some datasets. For example, in some datasets smoking was categorised as non-smoker/smoker, whereas more detailed measures would provide fuller adjustment. Again, similar results across datasets, despite differences in the likely extent of measurement error and potential for residual confounding, suggest that these issues have not importantly influenced results. The CPP data is much older than the other datasets. However, if there is a true causal effect of BMI on PTB, this would also be the case in older cohorts. Bearing in mind that most of the preterm births in CPP were spontaneous, the fact that the results for CPP showed the same pattern as in the more contemporary datasets supports the conclusion of a causal relationship. Lastly, we could not identify similar data in low- and middle-income countries (LMIC), where the use of electronic health records for clinical care is still limited and takes priority over their use for research [47]. Thus, our results may not generalise to LMIC populations.

## Conclusions

In summary, we have shown a consistent non-linear association between pre-pregnancy BMI and risk of PTB across different populations. Women starting pregnancy with a higher BMI appear to have a higher risk of PTB, but only through medically indicated deliveries. In contrast, women with lower BMI have an increased risk of both SPTB and MPTB. The overall patterns were similar among pregnancies in nulliparous women and those in parous women.

Women with overweight and obesity are monitored more frequently in most high-income countries due to the increased risk of MPTB due to pregnancy complications such as gestational diabetes and hypertension. Our findings suggest that consideration of the increased risk of SPTB in women with low BMI is also important and that advice to women planning a pregnancy, and clinicians supporting them, should consider both underweight and obesity as risks for PTB. Furthermore, given the wide-ranging and persistent impact of preterm birth across childhood [48], broader public health initiatives to optimise peri-conception maternal health through advice that highlights underweight as well as overweight could have profound population benefits.

### Supplementary Information

**Additional file 1: Supplementary text A.1.** Further details about the datasets and availability of confounders. **Supplementary text A.2.** Further details of statistical methods. **Supplementary Figure S1.** Summary of missing data. **Supplementary Table S1-S8.** For each dataset- characteristics of the whole sample, complete cases and excluded cases. **Supplementary Tables S9-S10.** Univariate risk of any PTB, SPTB and MPTB. **Supplementary Tables S11-S12.** Deviance from different fractional polynomials. **Supplementary Tables S13-S14.** Meta-analysis results. **Supplementary Tables S15-S16.** Adjusted odds ratios of any PTB, SPTB and MPTB by BMI category. **Supplementary Tables S17-S18.** Adjusted odds ratios for any very PTB, spontaneous very PTB and medically indicated very PTB by BMI category. **Supplementary Tables S19-S20.** Adjusted odds ratios for any PTB, SPTB and MPTB – inverse probability weighted. **Supplementary Tables S21-S22.** Adjusted odds ratios for any PTB, SPTB and MPTB excluding stillbirths. **Supplementary Tables S23-S24.** Adjusted odds ratios for any PTB, SPTB and MPTB excluding post term births. **Supplementary Tables S25-S26.** Adjusted odds ratios for any PTB, SPTB and MPTB excluding multiple births. **Supplementary Figures S1-S2.** Meta-analysis including and omitting CPP.

## Data Availability

CPP and NCHS Vital Statistics data are freely available (downloadable) at https://www.archives.gov/research/electronic-records/nih.html and https://www.cdc.gov/nchs/data_access/vitalstatsonline.htm, respectively. All proposals to use SAIL Databank data are subject to review by an independent Information Governance Review Panel (IGRP). Once approved, data access is via remote access to a privacy protecting safe haven. The SAIL application process is detailed at https://saildatabank.com/data/apply-to-work-with-the-data/. Access to CPRD data is subject to approval by the CPRD Research Data Governance (RDG) process (https://cprd.com/data-access). Our CPRD protocol was approved by the Independent Scientific Advisory Committee (ISAC; protocol number: 20_145R); approval via ISAC has now been replaced by the RDG process. Perinatal data for the BEBOLD platform are provided by the South Australian Department for Health and Wellbeing. Data are only accessible by researchers who have entered into an agreement with the Data Custodian and are approved by the SA Health Human Research Ethics Committee. The Danish linked data can be made available via remote access to a privacy protecting safe haven at Statistics Denmark after application to the Research Service Center at Statistics Denmark (forskningsservice@dst.dk). The process is detailed at Data for Research—Statistics Denmark (dst.dk). According to Norwegian legislation, individual-level registry data cannot be made publicly available. The data underlying this project can be accessed by direct application to the Directorate for E-Health (https://helsedata.no/en/) pending the required ethical approvals from the Regional Ethical Committees for Medical and Health Research of Norway. Access to NHS data from the Bradford Royal Infirmary is subject to regulations set out by the NHS Health Research Authority (https://www.hra.nhs.uk).

## Abbreviations

BEBOLD: Better evidence better outcomes linked data
BMI: Body mass index
BRI: Bradford royal infirmary
CPP: Collaborative perinatal project
CPRD: Clinical practice research datalink
HES: Hospital episode statistics
LMP: Last menstrual period
MBR: Medical birth registry
MBRN: Birth registry of norway
MID: Maternity indicators dataset
MPTB: Medically indicated preterm birth
NCCH: National community child health
NCHS: National center for health statistics
PTB: Preterm birth
SAIL: Secure anonymised information linkage
SPTB: Spontaneous preterm birth
