# Supplementary material for: Maternal pre-pregnancy body mass index and risk of preterm birth: a collaboration using large routine health datasets
Source: BMC Med. 2024 Jan 5;22:10. doi: 10.1186/s12916-023-03230-w (PMC10768428; doi:10.1186/s12916-023-03230-w)

Supplementary material (Maternal pre-pregnancy body mass index and risk of preterm birth: a collaboration using large routine health datasets)

A. Supplementary text

Supplementary text A.1: Further details about the datasets and availability of confounders

Collaborative Perinatal Project (CPP, USA)
This was a multisite, prospective cohort study carried out by the National Institute of Neurological and Communicative Disorders and Stroke. Gestational age at delivery was based on last menstrual period and was recorded in number of weeks, rounded to the nearest week (i.e. not completed weeks). Height was measured and maternal self-reported pre-pregnancy weight was recorded at enrolment (at the first antenatal visit for the majority of women: median; IQR gestational age: 21 weeks; 15-28 weeks); BMI was calculated from these. Maternal age, parity, ethnicity, education, and smoking was also measured at enrolment. At that time there were no ethics review boards but informed consent was obtained from the women.

Norwegian birth registry
The Birth Registry of Norway (MBRN) was established in 1967. Information on maternal self-reported pre-pregnancy height and weight was included in the notification form in the birth registry from 2008 onwards. However, the uptake of this additional registration took some time, resulting in a high proportion of missing in the first years. This information is recorded in antenatal records at 8-12 weeks gestation then later transcribed by midwives to the birth record. Estimates of gestational age at delivery was based on routine ultrasound measures taken at 18 weeks gestation, or last menstrual period for the small proportion of deliveries without ultrasound-based estimates (<5%). Maternal age, parity, country of birth and smoking was also registered at the time of delivery. No information was available in the Norwegian data on socio-economic position. The study was approved by the Regional Committee of Medical and Health Research Ethics of South/East Norway. The ethical committee provided a waiver of individual consent for this use of health register data for research in line with Norwegian legislation.

Danish linked data
All individuals living in Denmark are given a unique personal identification number, which can be used to identify individuals in all official registers. This personal identification number was used to link information about individuals from different registers covering the entire Danish population. From the Danish Medical Birth Registry, we identified all births in Denmark between 1 January 2004 and 31 December 2016. The study period started in 2004 as information on pre-pregnancy BMI was not registered until 2004. Information on BMI, gestational age, parity, maternal age, birth interval, smoking, multiple pregnancy, mode of delivery, and characteristics of labour (e.g. whether induced) was obtained from the Medical Birth Registry. Gestational age is based on routine ultrasound measures from early pregnancy or last menstrual period for a small proportion of pregnancies. Maternal pre-pregnancy BMI is calculated based on self-reported weight and height. Educational level and country of origin was obtained from population registers held by Statistics Denmark (https://www.dst.dk/en/TilSalg/Forskningsservice/Data). The processing and linkage of data were approved by the Danish Data Protection Agency (UCHP reference number: 514-0230/18-3000). Ethical approval or informed consent is not required for register-based studies in Denmark.

Clinical Practice Research Datalink (CPRD)
The CPRD GOLD data, including the pregnancy register, are sourced from a sub-sample of primary care centres from across the UK: from those practices that use Vision® GP software (https://cprd.com/sites/default/files/2022-11/2022-11%20CPRD%20GOLD%20Release%20Notes.pdf). These can be linked to a range of other health and

health-related datasets. Birth outcome (live or stillborn), delivery details, gestational age at delivery, and maternal age were obtained from the Hospital Episode Statistics (HES) maternity data (Copyright© 2020, re-used with the permission of NHS Digital. All rights reserved); parity and birth interval from the pregnancy register; and BMI (recorded in the data as BMI itself or derived from recorded height and weight) and smoking from the primary care data. Gestational age in completed weeks is derived from ultrasound or – if not available - last menstrual period. We required BMI to be measured a maximum of 12 months pre-pregnancy and/or a maximum of 15 weeks gestation. CPRD has National Research Ethics Service Committee (NRES) approval for research using the primary care and linked datasets. Individuals registered with participating GP practices are included in the CPRD dataset unless they specifically opt out. The CPRD study protocol was approved by the Independent Scientific Advisory Committee (ISAC; protocol number: 20_145R).

South Australian Better Evidence Better Outcomes Linked Data (BEBOLD) platform
Pregnancy data was obtained from the BEBOLD platform, which includes the South Australian Perinatal Statistics Collection 2007-2016. This is a mandatory collection of all births at least 400 grams or 20 weeks gestation. Maternal height and weight were reported at the first antenatal visit. Over 85% of women attend prior to 14 weeks pregnancy, with height and weight data not reported after 20 weeks gestation. Collection of height and weight data commenced in 2007, with a higher proportion of missing information in the first year of collection. Gestational age was determined from the first day of the last menstrual period if dates were reliable and early ultrasound (up to 20 weeks). A clinical examination could be used in the absence of these data or where there was uncertainty. Approval for the BEBOLD platform was obtained from the South Australian Department of Health's Human Research Ethics Committee, which included a waiver of individual consent for the use of de-identified administrative data.

US National Center for Health Statistics vital statistics (birth registration) data
US states are required to record births and deaths via certificates and Federal law mandates national collection and publication of these data. These data are compiled by the National Center for Health Statistics (NHCS), anonymised and made publicly available; they form the National Vital Statistics System. BMI was included in the fetal death data files from 2014 onwards and, at the time of analysis, data on fetal deaths were available up to the end of 2019. Each record in these datasets relates to a live birth or fetal death, rather than a pregnancy and there are no pregnancy or person-level identifiers in the dataset. To identify pregnancies we matched multiple births occurring close in time in which birth and maternal characteristics were the same. Gestational age at delivery was based on routine ultrasound measurements or last menstrual period for the small proportion (<1%) with no ultrasound-based data. Maternal pre-pregnancy weight and height were self-reported by the women at the time of birth. (When a mother registers a birth, she is required to complete a form – called the Mother's Worksheet - https://www.cdc.gov/nchs/data/dvs/moms-worksheet-2016.pdf. Information needed for the birth certificate is collected, as is additional socio-demographic – race, education level, marital status and so on – as well as other data, including smoking, height and pre-pregnancy weight.) These publicly available datasets are anonymised.

Welsh linked data: Secure Anonymised Information Linkage (SAIL) Databank
Linkable datasets from the SAIL Databank are made available for approved analyses via a secure research environment, the UK Secure Research Platform (SeRP). The datasets used for the current study were primary care records, the National Community Child Health (NCCH) Database NCCHD (birth registration plus child health and immunisation data), the Welsh Demographic Service Dataset (demographic characteristics of individuals registered with a GP in Wales) and the Maternity Indicators Dataset (MID) (data - from 2014 onwards - on women from their first antenatal assessment together with data on labour and birth). The MID and the NCCH datasets provided all variables except socio-economic position (Index of Multiple Deprivation (IMD) 2014, which came from the Welsh Demographic Service Dataset, and BMI, which either came from the MID (derived

from recorded height and weight) or the primary care data (recorded as BMI itself or derived from recorded height and weight). BMI was from the MID for 77% of pregnancies and from the GP data for the remainder. If BMI came from the primary care data, we required it to be measured a maximum of 12 months pre-pregnancy and/or a maximum of 15 weeks gestation. Gestational age at delivery is based on early ultrasound if available or – if not available – last menstrual period. Details regarding ethics and consent have been described previously; individuals are able to opt out of their data being transferred to SAIL.

Bradford maternity data (UK)
The Bradford Royal Infirmary is a large teaching hospital in Bradford, England, operated by the Bradford Teaching Hospitals NHS Foundation Trust. Gestational age at delivery was based on early ultrasound if available or – if not available – last menstrual period. BMI was derived from height and weight, which were measured by clinical staff at the first antenatal appointment. All data were anonymised and therefore patient consent and ethical approval was not required.

Availability of confounders
Socio-economic position (SEP) was measured differently across the datasets: years of education, index of multiple deprivation (IMD), Townsend score (another area-based deprivation index), and occupation. Ethnicity is not recorded in the Danish or Norwegian registries; in the Danish data, country of origin was included instead. We were able to adjust for either birth or pregnancy interval in all datasets except the Bradford maternity data; where a dataset had both birth and pregnancy interval, we used the most complete. Depending on the dataset, smoking was recorded as either (i) current smoker/non-smoker, (ii) ever/never smoked, or (iii) smoking during pregnancy, with information collected in each trimester (see Supplementary Tables S1-S8). Maternal age (in years) and parity (total number of previous births) were available and categorised in the same way in all datasets (maternal age categorised as: <25, 25-29, 30-34, 35-39, and 40+ years, although presented as mean (SD) years in Supplementary Tables S1-S8; parity was categorised as 0, 1, 2, 3, 4+).

Supplementary text A.2: Further details of statistical methods

a) Fractional polynomial models

To fit the fractional polynomials, BMI was first scaled [scaled BMI = (BMI-10)/5]. This is done because if the values of the variable are too large (or too small), this can generate extreme values with certain powers of this variable (e.g. cubic or squared reciprocal powers). Supplementary Table S10 gives the deviance for the different two-degree and three-degree fractional polynomials for each outcome in each dataset. Model fit was assessed using the change in deviance. Since the three-degree models fit better in all datasets except Connected Bradford and CPP and because there was more consistency in terms of the best-fitting three power models, we selected the optimal model from among those with three powers of scaled BMI. For all three outcomes – any preterm birth (PTB), spontaneous preterm birth (SPTB) and medically indicated PTB (MPTB), the optimal model had terms of scaled BMI of -2, -2, -2 (i.e. 1/scaled BMI^2, ln(scaled BMI) x 1/scaled BMI^2 and ln(scaled BMI) x ln(scaled BMI) x 1/scaled BMI^2). For any preterm birth, this polynomial was the best fitting model in three datasets and the second-best fitting in three; for SPTB it was the best fitting model in six of the eight datasets; and for medically indicated PTB it was the best fitting model in three datasets and the second-best in three.

b) Predicted risks and meta-analyses

For each dataset, the fitted (logistic) model was of the form:

$$\text{Log odds (PTB)} = \propto + (\beta_1 \times \text{sBMI}^{-2}) + \left(\beta_2 \times \ln(\text{sBMI}) \times \text{sBMI}^{-2}\right) + \left(\beta_3 \times \ln(\text{sBMI}) \times \ln(\text{sBMI}) \times \text{sBMI}^{-2}\right) + \boldsymbol{X}\gamma \quad [1]$$

where sBMI is scaled BMI, $\mathbf{X}$ represents the confounders, and γ the vector of coefficients for the confounders obtained from the logistic regression and with equivalent models for SPTB and MPTB. The study-specific estimates of the constant, α, and fractional polynomial terms, $\beta_1, \beta_2, \beta_3$, were used to plot the predicted risk of PTB against BMI for individuals in the reference category of all confounders for each study.

We used multivariate random effects meta-analysis to pool the fractional polynomial terms (i.e. the βs, the regression coefficients for the powers of sBMI) and the constant term, α, and then used the pooled estimates to plot the predicted (pooled) risk of PTB against BMI.

c) Obtaining the estimated BMI at which the risk of PTB is lowest

These were obtained by differentiating the function given in equation [1] with respect to (scaled) BMI – minimum points occur where the value of this differential is equal to zero.

To calculate confidence intervals, the estimates of α, β_1, β_2, and β_3, together with their variance covariance matrix were used to generate 100,000 bootstrapped samples of these coefficients using the drawnorm function in Stata and the value of BMI at which the minimum point occurred was calculated for each sample. The confidence limits were obtained by taking the 2.5th and 97.5th percentiles of these.

B. Supplementary tables and figures

Supplementary Figure S1: Summary of missing data

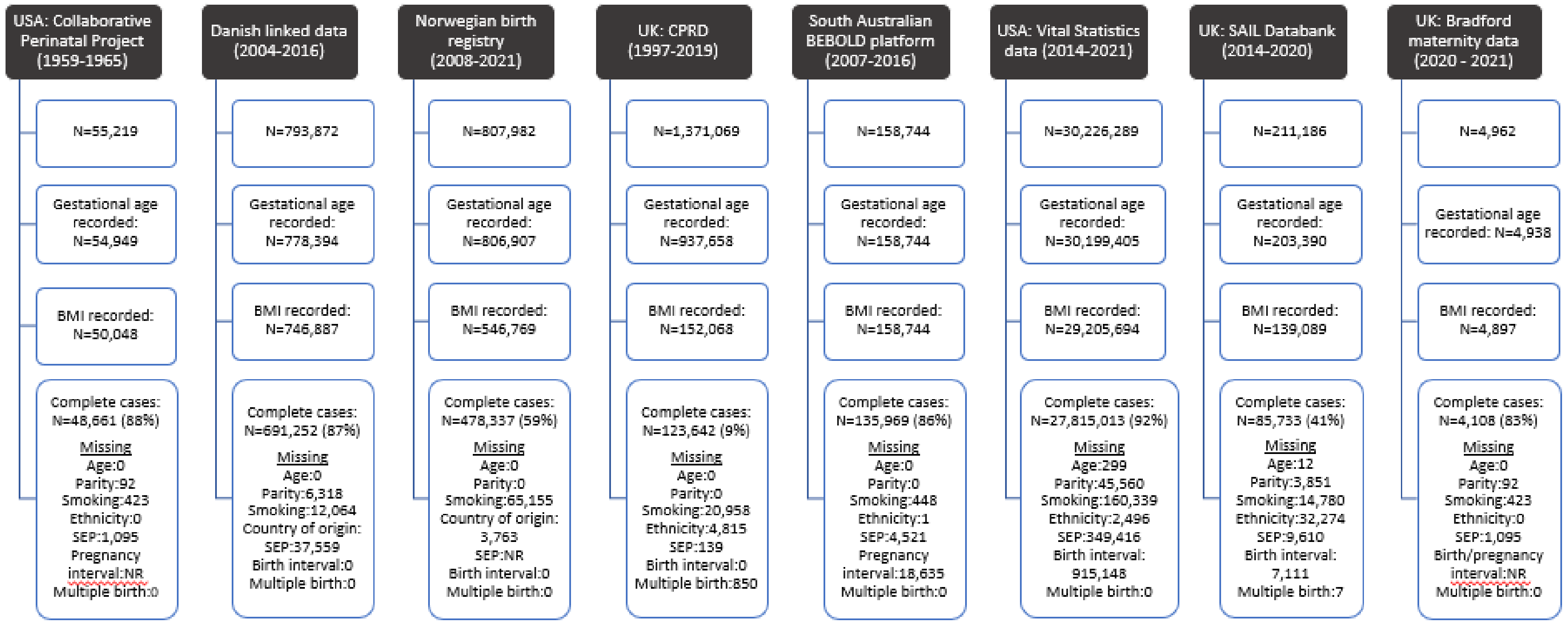

Note: Numbers missing on this figure are among those with recorded gestational age and BMI so do not necessarily match those shown in Supplementary Tables S1-S8

Supplementary Table S1: Characteristics of the whole sample, complete cases and excluded cases: Collaborative Perinatal Project (USA, 1959-1965)

Characteristic		Whole sample (N=55,219)	Complete cases (N=48,658)	Excluded cases (N=6,561)
		Frequency (%[1])	Frequency (%)	Frequency (%[1])
Mother's age (years)	Mean (SD)	24 (6.0)	24 (6.0)	
Parity	0	16,449 (29.9%)	14,642 (30.1%)	1,807 (28.3%)
	1	12,571 (22.8%)	11,108 (22.8%)	1,463 (22.9%)
	2	9,021 (16.4%)	7,943 (16.3%)	1,078 (16.9%)
	3	6,203 (11.3%)	5,456 (11.2%)	747 (11.7%)
	4+	10,793 (19.6%)	9,509 (19.5%)	1,284 (20.1%)
	Missing	182		182
Smoking status	Non-smoker	29,084 (53.3%)	26,071 (53.6%)	3,013 (50.6%)
	Smoker	25,531 (46.8%)	22,587 (46.4%)	2,944 (49.4%)
	Missing	604		604
Ethnicity	White	25,344 (45.9%)	21,249 (43.7%)	4,095 (62.4%)
	Black	25,778 (46.7%)	23,831 (50.0%)	1,947 (29.7%)
	Other[2]	4,097 (7.4%)	3,578 (7.4%)	519 (7.9%)
	Missing	0		0
SEP: Maternal education	< High school	31,138 (57.8%)	28,411 (58.4%)	2,727 (53.7%)
	High school	16,320 (30.3%)	14,701 (30.2%)	1,619 (31.3%)
	> High school	6,374 (11.8%)	5,546 (11.4%)	828 (16.0%)
	Missing	1,387		1,387
Pregnancy size	Singleton	54,584 (98.9%)	48,119 (98.9%)	6,465 (98.9%)
	Multiple	613 (1.1%)	539 (1.1%)	74 (1.1%)
	Missing	22		22
BMI (kg/m^2)	Mean (SD)	22.8 (4.3)	22.8 (4.3)	
BMI (kg/m^2)	<18.5	4,629 (9.2%)	4,479 (9.2%)	150 (9.3%)
	18.5-24.9	34,426 (68.5%)	33,325 (68.5%)	1,101 (68.3%)
	25-29.9	7,829 (15.6%)	7,594 (15.6%)	235 (14.6%)
	30-34.9	2,375 (4.7%)	2,296 (4.7%)	79 (4.9%)
	35+	1,011 (2.0%)	964 (2.0%)	47 (2.9%)
	Missing	4,949		4,949
Preterm	Yes	8,552 (15.6%)	7,365 (15.1%)	1,187 (18.9%)
	Missing	270		270

1. Percentages given in this column exclude those with missing data

Supplementary Table S2: Characteristics of the whole sample, complete cases and excluded cases: Danish linked data (2004-2016)

Characteristic		Whole sample (N=793,872)	Complete cases (N=691,252)	Excluded cases (N=102,620)
		Frequency (%[1])	Frequency (%)	Frequency (%[1])
Mother's age (years)	Mean (SD)	30 (5.0)	30 (4.9)	
Parity	0	351,964 (45.7%)	313,913 (45.4%)	38,051 (47.7%)
	1	281,193 (36.5%)	255,350 (36.9%)	25,843 (32.4%)
	2	102,281 (13.3%)	92,022 (13.3%)	10,259 (12.9%)
	3	24,513 (3.2%)	21,192 (3.1%)	3,321 (4.2%)
	4+	11,121 (1.4%)	8,775 (1.3%)	2,346 (2.9%)
	Missing	22,800		22,800
Smoking status	Never smoked	662,836 (86.7%)	598,908 (86.6%)	63,928 (86.8%)
	Stopped 1st trimester	17,654 (2.3%)	16,256 (2.4%)	1,398 (1.9%)
	Smoker	84,370 (11.0%)	76,088 (11.0%)	8,282 (11.3%)
	Missing	29,012		29,012
Country of origin	Denmark	656,860 (83.9%)	603,329 (87.3%)	53,531 (58.6%)
	Other western country	34,662 (4.4%)	23,468 (3.4%)	11,194 (12.3%)
	Non-western country	91,035 (11.6%)	64,455 (9.3%)	26,580 (29.1%)
	Missing	11,315		11,315
Birth interval	No previous birth	370,096 (46.6%)	310,670 (44.9%)	59,426 (57.9%)
	<12 months	2,852 (0.4%)	2,166 (0.3%)	606 (0.6%)
	12-23 months	73,505 (9.3%)	64,608 (9.4%)	8,897 (8.7%)
	24+ months	347,419 (43.8%)	313,808 (45.4%)	33,611 (32.8%)
	Missing	0		0
SEP: Maternal education	< Secondary	136,540 (18.3%)	123,598 (17.9%)	12,942 (22.9%)
	Secondary/post-secondary/short-cycle tertiary	328,425 (43.9%)	303,474 (43.9%)	24,951 (44.2%)
	Bachelor's degree/higher	282,720 (37.8%)	264,180 (38.2%)	18,540 (32.9%)
	Missing	46,187		46,187
Pregnancy size	Singleton	776,046 (97.8%)	675,542 (97.7%)	100,504 (97.9%)
	Multiple	17,826 (2.3%)	15,710 (2.3%)	2,116 (2.1%)
	Missing	0		0
BMI (kg/m^2)	Mean (SD)	24.3 (5.0)	24.3 (5.0)	
BMI (kg/m^2)	<18.5	32,325 (4.3%)	28,832 (4.2%)	3,493 (6.3%)
	18.5-24.9	465,602 (62.3%)	430,052 (62.2%)	35,550 (63.7%)
	25-29.9	157,415 (21.1%)	146,268 (21.2%)	11,147 (20.0%)
	30-34.9	60,264 (8.1%)	56,394 (8.2%)	3,870 (6.9%)
	35-39.9	21,468 (2.9%)	20,238 (2.9%)	1,230 (2.2%)
	40+	10,027 (1.3%)	9,468 (1.4%)	559 (1.0%)
	Missing	46,771		46,771
Preterm	Yes	46,946 (6.0%)	40,303 (5.8%)	6,643 (6.5%)
	Missing	15,478		15,478

1. Percentages given in this column exclude those with missing data

Supplementary Table S3: Characteristics of the whole sample, complete cases and excluded cases: Norwegian birth registry (2008-2021)

Characteristic		Whole sample (N=807,982)	Complete cases (N=478,337)	Excluded cases (N=329,645)
		Frequency (%[1])	Frequency (%)	Frequency (%[1])
Mother's age (years)	Mean (SD)	30 (5.1)	30 (5.0)	
Parity	0	342,524 (42.5%)	207,966 (43.5%)	134,558 (41.0%)
	1	295,726 (36.7%)	174,164 (36.4%)	121,562 (37.0%)
	2	120,367 (14.9%)	69,567 (14.5%)	50,800 (15.5%)
	3	32,265 (4.0%)	18,123 (3.8%)	14,142 (4.3%)
	4+	16,025 (2.0%)	8,517 (1.8%)	7,508 (2.3%)
	Missing	1,075		1,075
Smoking status	Never smoked	591,768 (86.5%)	417,648 (87.3%)	174,120 (84.7%)
	Smoked before pregnancy	46,129 (6.8%)	33,415 (7.0%)	12,714 (6.2%)
	Stopped early pregnancy	16,164 (2.4%)	9,519 (2.5%)	6,645 (3.2%)
	Smoked throughout pregnancy	29,740 (4.4%)	17,755 (3.7%)	11,985 (5.8%)
	Missing	124,181		124,181
Country of birth	Norway	586, 942 (73.3%)	352,145 (73.6%)	234,797 (72.8%)
	Other western country	43,054 (5.4%)	25,123 (5.3%)	17,931 (5.6%)
	Non-western country	170,962 (21.3%)	101,069 (21.1%)	69,893 (21.7%)
	Missing	7,024		7,024
Birth interval	No previous live birth	382,249 (47.4%)	230,173 (48.2%)	152,076 (46.3%)
	<12 months	53,311 (6.6%)	29,695 (6.2%)	23,616 (7.2%)
	12-23 months	127,055 (15.8%)	73,861 (15.4%)	53,194 (16.2%)
	24+ months	244,292 (30.3%)	144,608 (30.2%)	99,684 (30.3%)
	Missing	1,075		1,075
Pregnancy size	Singleton	793,934 (98.4%)	470,715 (98.4%)	323,219 (98.4%)
	Multiple	12,973 (1.6%)	7,622 (1.6%)	5,351 (1.6%)
	Missing	1,075		1,075
BMI (kg/m^2)	Mean (SD)	24.4 (4.7)	24.4 (4.7)	
BMI (kg/m^2)	<18.5	21,479 (3.9%)	18,541 (3.9%)	2,938 (4.3%)
	18.5-24.9	333,714 (61.0%)	291,525 (61.0%)	42,189 (61.7%)
	25-29.9	123,554 (22.6%)	108,318 (22.6%)	15,236 (22.3%)
	30-34.9	47,348 (8.7%)	41,701 (8.7%)	5,647 (8.3%)
	35-39.9	15,792 (2.9%)	13,942 (2.9%)	1,850 (2.7%)
	40+	4,882 (0.9%)	4,310 (0.9%)	572 (0.8%)
	Missing	261,213		261,213
Preterm	Yes	46,549 (5.8%)	25,604 (5.4%)	20,945 (6.4%)
	Missing	1,075		1,075

1. Percentages given in this column exclude those with missing data

Supplementary Table S4: Characteristics of the whole sample, complete cases and excluded cases: Clinical Practice Research Datalink (UK, 1997-2019)

Characteristic		Whole sample (N=1,371,069)	Complete cases (N=123,642)	Excluded cases (N=1,247,427)
		Frequency (%[1])	Frequency (%)	Frequency (%[1])
Mother's age (years)	Mean (SD)	29 (6.0)	30 (5.8)	
Parity	0	689,861 (50.3%)	62,871 (50.9%)	626,990 (50.3%)
	1	449,437 (32.8%)	43,882 (35.5%)	405,555 (32.5%)
	2	156,473 (11.4%)	12,359 (10.0%)	144,114 (11.6%)
	3	49,983 (3.7%)	3,328 (2.7%)	46,655 (3.7%)
	4+	25,315 (1.9%)	1,202 (1.0%)	24,113 (1.9%)
	Missing	0		0
Smoking status	Never smoked	460,522 (46.6%)	57,865 (46.8%)	402,657 (46.6%)
	Current/ex-smoker	527,956 (53.4%)	65,777 (53.2%)	462,179 (53.4%)
	Missing	382,591		382,591
Ethnicity	White	1,133,171 (84.8%)	103,975 (84.1%)	1,029,196 (84.9%)
	Black	60,732 (4.6%)	5,299 (4.3%)	55,433 (4.6%)
	Asian	91,352 (6.8%)	8,895 (7.2%)	82,457 (6.8%)
	Mixed/other	50,398 (3.8%)	5,473 (4.4%)	44,925 (3.7%)
	Missing	35,416		35,416
Birth interval	No previous birth	689,590 (50.3%)	62,830 (50.8%)	626,760 (50.2%)
	<12 months	21,596 (1.6%)	1,198 (1.0%)	20,398 (1.6%)
	12-23 months	162,209 (11.8%)	13,976 (11.3%)	148,233 (11.9%)
	24+ months	497,674 (36.3%)	45,638 (36.9%)	452,036 (36.2%)
	Missing	0		0
SEP: Townsend quintile	Least deprived	245,500 (17.9%)	22,268 (18.0%)	223,232 (18.4%)
	2	248,903 (18.2%)	22,265 (18.0%)	226,638 (18.7%)
	3	278,498 (20.3%)	25,172 (20.4%)	253,326 (20.9%)
	4	316,351 (23.1%)	28,862 (23.3%)	287,489 (23.7%)
	Most deprived	280,066 (20.5%)	25,075 (20.3%)	254,991 (21.0%)
	Missing	1,751		1,751
Pregnancy size	Singleton	1,139,837 (97.3%)	120,602 (97.5%)	1,019,235 (97.2%)
	Multiple	32,227 (2.8%)	3,040 (2.5%)	29,187 (2.8%)
	Missing	199,005		199,005
BMI (kg/m^2)	Mean (SD)	25.6 (5.8)	25.7 (5.8)	
BMI (kg/m^2)	<18.5	9,147 (4.3%)	5,237 (4.2%)	3,910 (4.4%)
	18.5-24.9	111,248 (52.3%)	63,774 (51.6%)	47,474 (53.3%)
	25-29.9	52,829 (24.8%)	30,750 (24.9%)	22,079 (24.8%)
	30-34.9	24,022 (11.3%)	14,438 (11.7%)	9,584 (10.8%)
	35-39.9	10,082 (4.7%)	6,222 (5.0%)	3,860 (4.3%)
	40+	5,330 (2.5%)	3,221 (2.6%)	2,109 (2.4%)
	Missing	1,158,411		1,158,411
Preterm	Yes	76,882 (8.2%)	10,760 (8.7%)	66,122 (8.1%)
	Missing	433,411		433,411

1. Percentages given in this column exclude those with missing data

Supplementary Table S5: Characteristics of the whole sample, complete cases and excluded cases: South Australian BEBOLD platform (2007-2016)

Characteristic		Whole sample (N=158,744)	Complete cases (N=135,969)	Excluded cases (N=22,775)
		Frequency (%[1])	Frequency (%)	Frequency (%[1])
Mother's age (years)	Mean (SD)	30 (6.0)	29 (5.5)	
Parity	0	67,835 (42.7%)	56,932 (41.9%)	10,903 (47.9%)
	1	54,911 (34.6%)	48,216 (35.5%)	6,695 (29.4%)
	2	22,669 (14.3%)	19,529 (14.4%)	3,140 (13.8%)
	3	7,981 (5.0%)	6,785 (5.0%)	1,196 (5.3%)
	4+	5,348 (3.4%)	4,507 (3.3%)	841 (3.7%)
	Missing	0		0
Smoking status	Non-smoker	131,639 (83.2%)	114,474 (84.2%)	17,165 (76.9%)
	Stopped before 1st antenatal appt	5,904 (3.7%)	4,751 (3.5%)	1,153 (5.2%)
	Current smoker	20,753 (13.1%)	16,744 (12.3%)	4,009 (18.0%)
	Missing	448		448
Ethnicity	Caucasian	125,500 (79.1%)	108,789 (80.0%)	16,711 (73.4%)
	Indigenous	5,150 (3.2%)	4,169 (3.1%)	981 (4.3%)
	Asian	18,916 (11.9%)	15,429 (11.4%)	3,487 (15.3%)
	Other	9,177 (5.8%)	7,582 (5.6%)	1,595 (7.0%)
	Missing	1		1
Pregnancy interval	No previous pregnancy	48,893 (34.9%)	46,980 (34.6%)	1,913 (46.2%)
	<12 months	7,388 (5.3%)	7,201 (5.3%)	187 (4.5%)
	12-23 months	29,032 (20.7%)	28,307 (20.8%)	725 (17.5%)
	24+ months	54,796 (39.1%)	53,481 (39.3%)	1,315 (31.8%)
	Missing	18,635		18,635
SEP: Occupation	Manager, professional, administrator	34,332 (22.3%)	30,964 (22.8%)	3,368 (18.5%)
	Para-professional, tradesperson, clerk, sales	58,621 (38.0%)	51,698 (38.0%)	6,923 (37.9%)
	Driver, labourer, plant/machine operator	5,415 (3.5%)	4,594 (3.4%)	821 (4.5%)
	Student, retired, unemployed, home duties	55,855 (36.2%)	48,713 (35.8%)	7,142 (39.1%)
	Missing	4,521		4,521
Pregnancy size	Singleton	154,542 (97.4%)	132,425 (97.4%)	22,117 (97.1%)
	Multiple	4,202 (2.7%)	3,544 (2.6%)	658 (2.9%)
	Missing	0		0
BMI (kg/m^2)	Mean (SD)	26.6 (6.2)	26.6 (6.2)	
BMI (kg/m^2)	<18.5	4,454 (2.8%)	3,801 (2.8%)	653 (2.9%)
	18.5-24.9	72,220 (45.5%)	61,941 (45.6%)	10,279 (45.1%)
	25-29.9	43,633 (27.5%)	37,351 (27.5%)	6,282 (27.6%)
	30-34.9	22,039 (13.9%)	18,858 (13.9%)	3,181 (14.0%)
	35-39.9	9,999 (6.3%)	8,582 (6.3%)	1,417 (6.2%)
	40+	6,399 (4.0%)	5,436 (4.0%)	963 (4.2%)
	Missing	0		0
Preterm	Yes	13,003 (8.2%)	10,832 (8.0%)	2,171 (9.5%)
	Missing	0		0

1. Percentages given in this column exclude those with missing data

Supplementary Table S6: Characteristics of the whole sample, complete cases and excluded cases: National Center for Health Statistics Vital Statistics Data (USA, 2014-2021)

Characteristic		Whole sample[1] (N=30,226,289)	Complete cases (N=27,815,013)	Excluded cases (N=2,411,276)
		Frequency (%[1])	Frequency (%)	Frequency (%[1])
Mother's age (years)	Mean (SD)	29 (6)	29 (6)	
Parity	0	11,724,134 (38.9%)	11,172,793 (40.2%)	551,341 (23.6%)
	1	9,588,930 (31.8%)	8,704,278 (31.3%)	884,652 (37.8%)
	2	5,079,579 (16.9%)	4,586,480 (16.5%)	493,099 (21.1%)
	3	2,193,648 (7.3%)	1,968,725 (7.1%)	224,923 (9.6%)
	4+	1,568,133 (5.2%)	1,382,737 (5.0%)	185,396 (7.9%)
		71,865		71,865
Smoking status	Non-smoker	27,198,266 (91.3%)	25,383,002 (91.3%)	1,815,264 (91.6%)
	Stopped early pregnancy	909,062 (3.1%)	864,551 (3.1%)	44,511 (2.2%)
	Smoked through pregnancy	1,690,463 (5.7%)	1,567,460 (5.6%)	123,003 (6.2%)
	Missing	428,498		428,498
Race/ ethnicity	White	22,200,095 (73.8%)	20,673,071 (74.3%)	1,527,024 (67.2%)
	Black	4,741,209 (15.8%)	4,284,583 (15.4%)	456,626 (20.1%)
	Native American/Alaskan	292,218 (1.0%)	267,646 (1.0%)	24,572 (1.1%)
	Asian	1,980,667 (6.6%)	1,787,928 (6.4%)	192,739 (8.5%)
	Native Hawaiian/Pacific Islander	99,879 (0.3%)	83,076 (0.3%)	16,803 (0.7%)
	Mixed race	774,958 (2.6%)	718,709 (2.6%)	56,249 (2.5%)
	Missing	137,263		137,263
(Live) birth interval	No previous live birth	11,725,728 (40.3%)	11,172,793 (40.2%)	552,935 (43.2%)
	<12 months	249,523 (0.9%)	236,130 (0.9%)	13,393 (1.0%)
	12-23 months	3,686,546 (12.7%)	3,527,959 (12.6%)	158,587 (12.4%)
	24+ months	13,432,607 (46.2%)	12,878,131 (46.3%)	554,476 (43.3%)
	Missing	1,131,885		1,131,885
SEP: Maternal education	< High school	3,903,303 (13.2%)	3,576,680 (12.9%)	326,623 (18.2%)
	High school	7,625,126 (25.8%)	7,133,632 (25.7%)	491,494 (27.4%)
	College, no degree	8,442,331 (28.5%)	7,971,074 (28.7%)	471,257 (26.3%)
	Degree/higher	9,636,486 (32.6%)	9,133,627 (32.8%)	502,859 (28.1%)
	Missing	619,043		619,043
Pregnancy size	Singleton	29,701,333 (98.3%)	27,358,755 (98.4%)	2,342,578 (97.2%)
	Multiple	524,956 (1.7%)	456,258 (1.6%)	68,698 (2.8%)
	Missing	0		0
BMI (kg/m^2)	Mean (SD)	27.1 (6.7)	27.1 (6.7)	
BMI (kg/m^2)	<18.5	955,566 (3.3%)	909,172 (3.3%)	46,394 (3.3%)
	18.5-24.9	12,733,325 (43.6%)	12,135,585 (43.6%)	597,740 (42.4%)
	25-29.9	7,686,166 (26.3%)	7,302,262 (26.3%)	383,904 (27.2%)
	30-34.9	4,166,717 (14.3%)	3,958,840 (14.2%)	207,877 (14.7%)
	35-39.9	2,149,445 (7.4%)	2,047,066 (7.4%)	102,379 (7.3%)
	40+	1,533,232 (5.3%)	1,462,088 (5.3%)	71,144 (5.0%)
	Missing	1,001,838		1,001,838
Preterm	Yes	2,793,509 (9.3%)	2,494,570 (9.0%)	298,939 (12.5%)
	Missing	26,884		26,884

1. Percentages given in this column exclude those with missing data

Supplementary Table S7: Characteristics of the whole sample, complete cases and excluded cases: Secure Anonymised Information Linkage (SAIL) Databank (Wales, UK, 2014-2020)

Characteristic		Whole sample (N=211,186)	Complete cases (N=85,733)	Excluded cases (N=125,453)
		Frequency (%[2])	Frequency (%)	Frequency (%[2])
Mother's age (years)	Mean (SD)	29 (5.7)	29 (5.7)	
Parity	0	76,108 (39.7%)	33,590 (39.2%)	42,518 (40.1%)
	1	70,109 (36.5%)	29,998 (35.0%)	40,111 (37.8%)
	2	30,278 (15.8%)	14,774 (17.2%)	15,504 (14.6%)
	3	9,795 (5.1%)	4,831 (5.6%)	4,964 (4.7%)
	4+	5,574 (2.9%)	2,540 (3.0%)	3,034 (2.9%)
		19,322		19,322
Smoking status	Non-smoker	123,533 (78.5%)	67,143 (78.3%)	56,390 (78.7%)
	Smoker	33,849 (21.5%)	18,590 (21.7%)	15,259 (21.3%)
	Missing	53,804		53,804
Ethnicity	White	140,252 (88.2%)	77,304 (90.2%)	62,948 (85.9%)
	Black	4,812 (3.0%)	1,768 (2.1%)	3,044 (4.2%)
	Asian	5,859 (3.7%)	2,725 (3.2%)	3,134 (4.3%)
	Mixed race	8,103 (5.1%)	3,936 (4.6%)	4,167 (5.7%)
	Missing	52,160		52,160
Birth interval	No previous birth	76,108 (39.4%)	33,590 (39.2%)	42,518 (39.7%)
	<12 months	2,169 (1.1%)	838 (1.0%)	1,331 (1.2%)
	12-23 months	21,948 (11.4%)	9,868 (11.5%)	12,080 (11.3%)
	24+ months	92,572 (48.1%)	41,437 (48.3%)	51,135 (47.8%)
	Missing	18,389		18,389
IMD quintile	Least deprived	29,351 (15.7%)	13,637 (15.9%)	15,714 (15.5%)
	2	31,169 (16.6%)	13,142 (15.3%)	18,027 (17.7%)
	3	36,747 (19.6%)	16,257 (19.0%)	20,490 (20.2%)
	4	40,900 (21.8%)	19,340 (22.6%)	21,560 (21.2%)
	Most deprived	49,165 (26.2%)	23,357 (27.2%)	25,808 (25.4%)
	Missing	23,854		23,854
Pregnancy size	Singleton	205,417 (97.9%)	83,872 (97.8%)	121,545 (97.9%)
	Multiple	4,467 (2.1%)	1,861 (2.2%)	2,606 (2.1%)
	Missing	1,302		1,302
BMI (kg/m^2)	Mean (SD)	27.1 (6.4)	27.3 (6.4)	
BMI (kg/m^2)	<18.5	3,495 (2.5%)	1,932 (2.3%)	1,563 (2.9%)
	18.5-24.9	58,727 (42.0%)	35,041 (40.9%)	23,686 (43.9%)
	25-29.9	39,673 (28.4%)	24,564 (28.7%)	15,109 (28.0%)
	30-34.9	21,269 (15.2%)	13,657 (15.9%)	7,612 (14.1%)
	35-39.9	10,276 (7.4%)	6,601 (7.7%)	3,675 (6.8%)
	40+	6,305 (4.5%)	3,938 (4.6%)	2,367 (4.4%)
		71,441		71,441
Preterm	Yes	15,115 (7.4%)	6,180 (7.2%)	8,935 (7.6%)
		7,796		7,796

1. Percentages given in this column exclude those with missing data

Supplementary Table S8: Characteristics of the whole sample, complete cases and excluded cases: Bradford (UK, 2020-2021)

		Whole sample (N=4,962)	Complete cases (N=4,108)	Excluded case (N=854)
		Frequency (%[2])	Frequency (%)	Frequency (%[2])
Mother's age (years)	Mean (SD)	29 (5.6)	29 (5.6)	
Parity	0	2,292 (46.2%)	1,814 (44.2%)	478 (56.0%)
	1	1,481 (29.9%)	1,226 (29.8%)	255 (29.9%)
	2	708 (14.3%)	627 (15.3%)	81 (9.5%)
	3	289 (5.8%)	265 (6.5%)	24 (2.8%)
	4+	192 (3.9%)	176 (4.3%)	16 (1.9%)
	Missing	0		0
Smoking status	Non smoker	3,895 (78.6%)	3,194 (77.8%)	701 (82.7%)
	Smoker	1,061 (21.4%)	914 (22.3%)	147 (17.3%)
	Missing	6		6
Ethnicity	White	1,763 (42.1%)	1,702 (41.4%)	61 (75.3%)
	South Asian	1,930 (46.1%)	1,916 (46.6%)	14 (17.3%)
	Other	496 (11.8%)	490 (11.9%)	6 (7.4%)
	Missing	773		773
Pregnancy size	Singleton	4,893 (98.6%)	4,056 (98.7%)	837 (98.0%)
	Multiple	69 (1.4%)	52 (1.3%)	17 (2.0%)
	Missing	0		0
BMI (kg/m^2)	Mean (SD)	29.3 (6.2)	29.3 (6.3)	
BMI (kg/m^2)	<18.5	77 (1.6%)	68 (1.7%)	9 (1.1%)
	18.5-24.9	1,187 (24.1%)	999 (24.3%)	188 (23.1%)
	25-29.9	1,642 (33.4%)	1,358 (33.1%)	284 (34.9%)
	30-34.9	1,194 (24.3%)	989 (24.1%)	205 (25.2%)
	35-39.9	537 (10.9%)	453 (11.0%)	84 (10.3%)
	40+	284 (5.8%)	241 (5.9%)	43 (5.3%)
	Missing	41		41
Preterm	Yes	363 (7.4%)	290 (7.1%)	73 (8.8%)
	Missing	24		24

1. Percentages given in this column exclude those with missing data

Supplementary Table S9: Univariate risk of any preterm, spontaneous preterm and medically indicated preterm birth by BMI category – nulliparous women

BMI category	CPP, USA	Danish linked data	Norwegian birth registry	CPRD, UK	South Australian BEBOLD	US Vital Statistics data	SAIL Databank, UK	Bradford, UK
**Any PTB**								
<18.5	291 (17.1%)	1,154 (8.1%)	621 (6.6%)	303 (9.8%)	203 (10.1%)	43,002 (9.5%)	80 (9.4%)	7 (20.6%)
18.5-24.9	1,549 (13.8%)	14,217 (7.0%)	7,670 (5.8%)	3,057 (8.5%)	1,193 (8.0%)	431,647 (8.0%)	1,057 (7.0%)	39 (8.0%)
25-29.9	199 (14.9%)	4,605 (7.5%)	2,866 (6.7%)	1,349 (9.4%)	1,266 (8.5%)	249,984 (9.0%)	683 (7.3%)	35 (5.9%)
30-34.9	38 (12.2%)	1,897 (8.3%)	1,219 (7.6%)	631 (10.4%)	665 (10.0%)	147,091 (10.7%)	364 (7.5%)	25 (5.9%)
35-39.9	10 (10.6%)[1]	680 (8.4%)	426 (8.0%)	257 (10.8%)	322 (10.9%)	85,465 (12.2%)	209 (9.2%)	12 (6.6%)
40+		298 (8.0%)	122 (7.5%)	124 (10.4%)	179 (10.0%)	70,578 (14.1%)	102 (8.2%)	11 (12.0%)
All	2,087 (14.3%)	22,851 (7.3%)	12,924 (6.2%)	5,721 (9.1%)	4,928 (8.7%)	1,027,767 (9.2%)	2,495 (7.4%)	129 (7.1%)
**SPTB**								
<18.5	269 (16.0%)	603 (4.4%)	422 (4.6%)	190 (6.3%)	114 (5.9%)	24,515 (5.6%)	44 (5.4%)	6 (17.7%)
18.5-24.9	1,402 (12.7%)	6,993 (3.6%)	4,950 (3.8%)	1,950 (5.6%)	1,233 (4.5%)	219,863 (4.3%)	559 (3.8%)	25 (5.2%)
25-29.9	175 (13.3%)	2,035 (3.4%)	1,648 (4.0%)	780 (5.7%)	596 (4.2%)	112,475 (4.3%)	340 (3.8%)	21 (3.5%)
30-34.9	29 (9.6%)	755 (3.5%)	659 (4.3%)	309 (5.4%)	297 (4.7%)	60,186 (4.7%)	158 (3.4%)	12 (2.8%)
35-39.9	9 (9.7%)[1]	244 (3.2%)	205 (4.0%)	119 (5.3%)	125 (4.6%)	31,817 (4.9%)	84 (3.9%)	6 (3.3%)
40+		102 (2.9%)	68 (4.3%)	64 (5.7%)	80 (4.7%)	23,181 (5.1%)	33 (2.8%)	6 (6.5%)
All	1,884 (13.1%)	10,732 (3.6%)	7,952 (3.9%)	3,412 (5.6%)	2,445 (4.5%)	472,037 (4.5%)	1,218 (3.8%)	76 (4.2%)
**MPTB**								
<18.5	13 (0.9%)	551 (4.0%)	199 (2.2%)	112 (3.8%)	89 (4.7%)	17,940 (4.2%)	36 (4.4%)	
18.5-24.9	93 (1.0%)	7,224 (3.7%)	2,720 (2.1%)	1,088 (3.2%)	1,060 (3.9%)	205,902 (4.0%)	497 (3.4%)	15 (2.9%)[3]
25-29.9	13 (1.1%)	2,570 (4.3%)	1,218 (3.0%)	560 (4.1%)	670 (4.7%)	133,811 (5.1%)	338 (3.8%)	14 (2.4%)
30-34.9	<10[2]	1,142 (5.2%)	560 (3.6%)	315 (5.5%)	368 (5.8%)	84,650 (6.5%)	206 (4.4%)	13 (3.1%)
35-39.9	<5[2]	436 (5.6%)	221 (4.3%)	138 (6.1%)	197 (7.0%)	52,441 (7.9%)	125 (5.7%)	6 (3.3%)
40+		196 (5.4%)	54 (3.4%)	59 (5.2%)	99 (5.8%)	46,290 (9.7%)	69 (5.7%)	5 (5.4%)
All	127 (1.0%)	12,119 (4.0%)	4,972 (2.5%)	2,272 (3.8%)	2,483 (4.6%)	541,034 (5.0%)	1,271 (3.9%)	53 (2.9%)

1. BMI ≥35 kg/m^2 as too few individuals with BMI of 40 or higher in this dataset
2. Exact number suppressed for disclosure control purposes
3. BMI <25 kg/m^2 – groups combined for disclosure control purposes

Supplementary Table S10: Univariate risk of any preterm, spontaneous preterm and medically indicated preterm birth by BMI category – parous women

BMI category	CPP, USA	Danish linked data	Norwegian birth registry	CPRD, UK	South Australian BEBOLD	US Vital Statistics data	SAIL Databank, UK	Bradford, UK
**Any PTB**								
<18.5	510 (18.4%)	950 (6.6%)	551 (6.0%)	267 (12.5%)	213 (11.9%)	52,447 (11.5%)	127 (11.8%)	9 (26.5%)
18.5-24.9	3,483 (15.7%)	9,721 (4.3%)	6,854 (4.3%)	2,276 (8.1%)	2,372 (7.1%)	536,585 (7.9%)	1,434 (7.2%)	64 (12.5%)
25-29.9	872 (13.9%)	3,956 (4.7%)	3,129 (4.8%)	1,278 (7.8%)	1,504 (6.7%)	380,359 (8.4%)	1,001 (6.6%)	38 (5.0%)
30-34.9	298 (15.0%)	1,740 (5.2%)	1,438 (5.6%)	701 (8.4%)	982 (8.0%)	245,005 (9.5%)	594 (6.7%)	26 (4.6%)
35-39.9	115 (13.2%)[1]	690 (5.7%)	550 (6.4%)	328 (8.5%)	514 (9.1%)	138,878 (10.3%)	316 (7.3%)	14 (5.2%)
40+		395 (6.9%)	158 (5.9%)	189 (9.3%)	319 (8.8%)	113,529 (11.8%)	214 (8.0%)	10 (6.7%)
All	5,278 (15.5%)	17,452 (4.6%)	12,680 (4.7%)	5,039 (9.3%)	5,904 (7.5%)	1,466,803 (8.8%)	3,686 (7.1%)	161 (7.0%)
**SPTB**								
<18.5	455 (16.7%)	447 (3.2%)	362 (4.0%)	175 (8.6%)	136 (7.9%)	31,200 (7.2%)	74 (7.2%)	6 (17.7%)
18.5-24.9	2,986 (13.8%)	3,989 (1.8%)	4,004 (2.5%)	1,357 (5.0%)	1,266 (3.9%)	271,130 (4.2%)	721 (3.7%)	34 (6.6%)
25-29.9	743 (12.1%)	1,360 (1.7%)	1,654 (2.6%)	665 (4.2%)	697 (3.2%)	163,381 (3.8%)	432 (3.0%)	13 (1.7%)
30-34.9	250 (12.9%)	544 (1.7%)	693 (2.8%)	321 (4.0%)	416 (3.6%)	93,028 (3.8%)	220 (2.6%)	10 (1.8%)
35-39.9	87 (10.3%)[1]	204 (1.7%)	271 (3.3%)	147 (4.0%)	188 (3.5%)	46,070 (3.7%)	109 (2.6%)	13 (3.1%)[2]
40+		112 (2.1%)	78 (3.0%)	78 (4.1%)	113 (3.3%)	30,496 (3.5%)	72 (2.8%)	
All	4,521 (13.6%)	6,656 (1.8%)	7,062 (3.0%)	2,743 (4.7%)	2,816 (3.7%)	635,305 (4.0%)	1,628 (3.3%)	76 (3.3%)
**MPTB**								
<18.5	38 (1.7%)	503 (3.6%)	189 (2.2%)	92 (4.7%)	77 (4.7%)	20,772 (4.9%)	52 (5.2%)	
18.5-24.9	352 (1.9%)	5,732 (2.6%)	2,850 (1.8%)	905 (3.4%)	1,106 (3.5%)	259,242 (4.0%)	708 (3.7%)	33 (6.0%)[3]
25-29.9	82 (1.5%)	2,596 (3.1%)	1,475 (2.3%)	605 (3.9%)	807 (3.7%)	212,116 (4.9%)	566 (3.8%)	25 (3.3%)
30-34.9	36 (2.1%)	1,196 (3.6%)	745 (3.0%)	374 (4.6%)	566 (4.8%)	148,815 (6.0%)	371 (4.3%)	16 (2.9%)
35-39.9	20 (2.6%)[1]	486 (4.1%)	279 (3.4%)	179 (4.8%)	326 (6.0%)	91,067 (7.0%)	204 (4.8%)	11 (2.6%)[2]
40+		283 (5.0%)	80 (3.1%)	110 (5.6%)	206 (5.8%)	81,623 (8.8%)	141 (5.4%)	
All	528 (1.8%)	10,796 (2.9%)	5,618 (2.1%)	2,265 (3.9%)	3,088 (4.1%)	813,635 (5.1%)	2,042 (4.0%)	

1. BMI ≥35 kg/m^2 as too few individuals with BMI of 40 or higher in this dataset
2. BMI ≥35 kg/m^2 – groups combined for disclosure control purposes
3. BMI <25 kg/m^2 – groups combined for disclosure control purposes

Supplementary Table S11: Deviance from different fractional polynomials for each dataset – nulliparous women

	Polynomial terms (two-degree models)						Polynomial terms (three-degree models)					
**Any PTB**	**0.5 0.5**	**-0.5 -0.5**	**0, 0**	**-1 -1**			**-2 -2 -2**	**-2 -2 -1**	**0 0.5 0.5**	**1 2 2**		
CPP	11,376	11,375	11,376	**11,375**[*]			**11,373**[b]	11,373	11,376	11,375		
Danish	153,240	**153,214**[a]	153,225	153,221			**153,185**[b]	153,194	153,194	153,209		
Norwegian	**90,481**[a]	90,527	90,496	90,538			90,450	90,452	90,481	**90,444**[b]		
CPRD	36,593	36,592	**36,589**[a]	36,600			**36,572**[b]	36,575	36,585	36,584		
South Australian BEBOLD	30,065	30,049	30,057	**30,044**[a]			30,044	30,044	**30,038**[b]	30,054		
US Vital Statistics	6,436,193	**6,434,224**[a]	6,434,949	6,435,065			6,433,816	**6,433,659**[b]	6,433,855	6,435,026		
SAIL Databank	16,978	16,979	**16,976**[a]	16,984			**16,970**[b]	16,971	16,976	16,976		
Bradford	**873**[a]	875	874	876			874	873	873	**872**[*]		
**SPTB**	**0.5 0.5**	**-2 -2**	**-0.5 -0.5**	**-1 -1**			**-2 -2 -2**	**0.5 1 1**	**0 0.5 0.5**			
CPP	10,545	**10,543**[a]	10,545	10,544			**10,543**[*]	10,545	10,545			
Danish	90,382	**90,377**[a]	90,378	90,379			**90,372**[b]	90,374	90,375			
Norwegian	**63,939**[a]	63,946	63,954	63,951			**63,926**[b]	63,934	63,939			
CPRD	25,451	**25,449**[a]	25,451	25,451			**25,447**[b]	25,449	25,450			
South Australian BEBOLD	18,648	18,643	18,642	**18,641**[a]			18,640	18,640	**18,639**[b]			
US Vital Statistics	3,773,207	3,733,424	**3,772,679**[a]	3,772,784			3,772,459	**3,772,433**[b]	3,772,505			
SAIL Databank	10,108	**10,106**[a]	10,107	10,107			**10,105**[b]	10,106	10,107			
Bradford	**604**[*]	605	605	605			604	**603**[*]	603			
**MPTB**	**-0.5 -0.5**	**2 3**	**3 3**	**0 0**	**-0.5 -2**		**-2 -2 -2**	**-2 -2 -1**	**1 2 2**	**0 0.5 0.5**	**-2 -2 -0.5**	**0.5 1 1**
US CPP	1,406	1,404	**1,404**[a]	1,406	1,406		1,405	1,405	**1,403**[b]	1,405	1,405	1,404
Danish	**91,639**[a]	91,645	91,640	91,651	91,658		91,612	91,620	91,622	91,614	91,626	**91,608**[b]
Norwegian	42,483	42,451	**42,444**[a]	42,463	42,450		42,436	42,439	**42,429**[b]	42,457	42,441	42,443
CPRD	18,059	**18,056**[a]	18,059	18,058	18,065		**18,035**[b]	18,040	18,048	18,050	18,044	18,040
South Australian BEBOLD	17,407	17,414	17,414	17,413	**17,401**[a]		17,401	17,401	17,408	**17,395**[b]	17,401	17,398
US Vital Statistics	**3,846,349**[a]	3,847,457	3,847,705	3,847,123	3,847,597		3,846,029	**3,845,917**[b]	3,847,042	3,846,029	3,846,036	3,846,218
SAIL Databank	10,049	10,048	10,048	**10,045**[a]	10,057		10,043	10,042	10,044	10,045	**10,042**[b]	10,043
Bradford	436	436	436	**436**[*]	437		**434**[b]	434	435	435	434	436

a Best fitting two-degree model; b Best fitting three-degree model; *Best fitting (two/three-degree) model among those displayed. The best fitting models in CPP and Connected Bradford were not necessarily the best fitting in any other dataset. However, the deviance for the models displayed were very similar (or the same to the nearest whole number) to the deviance for the best-fitting model. CPP: Any PTB 11,373 (two-degree); SPTB 10,543 (three-degree); Connected Bradford: Any PTB 871 (three-degree); SPTB 604 (two-degree), 603 (three-degree); MPTB 436 (two-degree).

Supplementary Table S12: Deviance from different fractional polynomials for each dataset – parous women

	Polynomial terms (two-degree models)				Polynomial terms (three-degree models)			
**Any PTB**	**0.5 0.5**	**0 0.5**	**-0.5 -0.5**	**0 -0.5**	**-2 -2 -2**	**-2 -2 -1**	**0.5 1 1**	
CPP	**28,082**[*]	28,083	28,083	28,082	28,082	28,082	**28,081**[*]	
Danish	131,743	131,725	131,715	**131,709**[a]	**131,663**[b]	131,669	131,693	
Norwegian	95,534	**95,530**[a]	95,596	95,562	95,498	**95,494**[b]	95,510	
CPRD	**33,161**[a]	33,161	33,180	33,173	**33,149**[b]	33,150	33,159	
South Australian BEBOLD	40,037	40,028	**40,013**[a]	40,016	40,002	40,004	**40,001**[b]	
US Vital Statistics	9,286,981	9,286,016	9,284,914	**9,284,872**[a]	9,284,276	**9,284,033**[b]	9,284,797	
SAIL Databank	**24,890**[a]	24,893	24,908	24,905	**24,877**[b]	24,877	24,890	
Bradford	1,049	**1,048**[*]	1,049	1,048	1,050	1,049	**1,047**[*]	
**SPTB**	**0.5 0.5**	**-2 -2**	**0 -0.5**		**-2 -2 -2**	-2 -2 -1	1 1 1	**-2 -2 3**
CPP	**24,932**[*]	24,935	24,934		24,932	24,932	**24,930**[*]	24,935
Danish	63,406	63,424	63,401		63,387	**63,386**[b]	63,398	63,405
Norwegian	**61,475**[a]	61,526	61,516		**61,449**[a]	61,450	61,465	61,475
CPRD	21,278	**21,278**[a]	21,287		21,277	21,277	21,279	**21,276**[b]
South Australian BEBOLD	23,631	23,627	**23,625**[a]		23,621	23,623	**23,621**[b]	23,626
US Vital Statistics	5,081,324	**5,080,548**[a]	5,080,743		**5,080,450**[b]	5,080,485	5,080,519	5,080,536
SAIL Databank	13,452	**13,445**[a]	13,459		**13,442**[b]	13,442	13,450	13,443
Bradford	**595**[*]	596	596		**591**[*]	591	593	595
**MPTB**	**0 0.5**	**0 0**	**-0.5 -1**	**-0.5 -0.5**	**-2 -2 -2**	**-2 -2 -1**	**0.5 0.5 1**	
CPP	5,183	5,183	**5,183**[*]	5,183	5,182	**5,182**[*]	5,183	
Danish	90,232	90,223	90,224	**90,218**[a]	**90,187**[b]	90,193	90,202	
Norwegian	50,457	**50,456**[a]	50,488	50,476	**50,436**[b]	50,437	50,448	
CPRD	**18,099**[a]	18,100	18,113	18,109	**18,089**[b]	18,091	18,097	
South Australian BEBOLD	24,233	24,217	**24,217**[a]	24,218	24,208	24,211	**24,207**[b]	
US Vital Statistics	5,896,696	5,896,152	5,895,851	**5,895,576**[a]	5,895,466	**5,895,193**[b]	5,895,616	
SAIL Databank	**16,028**[a]	16,028	16,034	16,032	**16,021**[b]	16,022	16,047	
Bradford	**676**[*]	676	676	676	676	676	**676**[*]	

a Best fitting two-degree model; b Best fitting three-degree model; *Best fitting (two/three-degree) model among those displayed. The best fitting models in CPP and Connected Bradford were not necessarily the best fitting in any other dataset. However, the deviance for the models displayed were very similar (or the same to the nearest whole number) to the deviance for the best-fitting model. CPP: Any PTB 28,081 (two-degree), 28,080 (three-degree); SPTB 24,930 (both two- and three-degree); MPTB 5,182 (both three- and two-degree). Connected Bradford: Any PTB 1,048 (two-degree), 1,045 (three-degree); SPTB 595 (two-degree), 591 (three-degree); MPTB 676 (two-degree), 675 (three-degree).

Supplementary Table S13: Random effects meta-analysis: results are the terms (and their standard errors) for each dataset, the pooled result, and the weights (sBMI=scaled BMI) – nulliparous women

	Term				
Dataset	Constant	$sBMI^{-2}$	Ln(sBMI) × $sBMI^{-2}$	Ln(sBMI) × Ln(sBMI) × $sBMI^{-2}$	Weights (%)[1]
**Any PTB**					
CPP	-2.59 (0.27)	0.58 (0.22)	0.51 (0.67)	-0.74 (1.23)	11.8, 11.9, 7.3, 12.2
Danish	-2.16 (0.06)	0.09 (0.08)	-0.93 (0.21)	-3.27 (0.44)	13.9, 14.2, 17.3, 14.4
Norwegian	-2.04 (0.10)	-0.20 (0.18)	-1.49 (0.45)	-4.79 (0.86)	13.5, 14.2, 12.0, 12.9
CPRD	-1.98 (0.11)	-0.008 (0.13)	-1.35 (0.34)	-3.46 (0.71)	13.5, 13.6, 11.3, 13.4
South Australian BEBOLD	-2.10 (0.12)	0.51 (0.21)	-1.99 (0.55)	-3.10 (1.01)	13.3, 13.2, 8.7, 12.4
US Vital Statistics	-1.55 (0.008)	-0.45 (0.02)	-1.47 (0.04)	-5.91 (0.07)	14.3, 14.7, 32.2, 16.1
SAIL Databank	-2.32 (0.13)	0.55 (0.20)	-1.35 (0.44)	-2.90 (0.78)	13.4, 13.3, 8.9, 13.7
Bradford	-1.39 (0.90)	-1.18 (3.17)	10.3 (8.6)	-19.8 (11.5)	6.5, 5.4, 2.4, 5.0
**Pooled**	-2.10 (0.16)	0.14 (0.17)	-1.23 (0.11)	-3.59 (0.70)	-
**I^2**	97%	94%	36%	91%	-
**SPTB**					
CPP	-2.94 (0.27)	0.76 (0.22)	0.98 (0.71)	-0.22 (1.12)	10.7, 11.3, 9.6, 12.3
Danish	-3.27 (0.09)	0.53 (0.11)	0.49 (0.27)	-1.31 (0.57)	15.3, 15.5, 15.6, 15.0
Norwegian	-2.94 (0.12)	0.42 (0.20)	-0.65 (0.47)	-2.45 (0.97)	13.9, 14.7, 13.7, 11.3
CPRD	-3.00 (0.12)	0.41 (0.14)	-0.11 (0.37)	-0.87 (0.61)	15.0, 14.4, 13.6, 17.1
South Australian BEBOLD	-2.98 (0.19)	0.65 (0.43)	0.31 (1.13)	-3.37 (1.88)	11.8, 12.6, 10.0, 8.2
US Vital Statistics	-2.88 (0.01)	0.21 (0.02)	-0.15 (0.05)	-2.78 (0.10)	17.4, 17.3, 26.6, 19.1
SAIL Databank	-3.65 (0.18)	0.97 (0.28)	0.30 (0.60)	-0.61 (0.91)	13.7, 12.1, 9.2, 15.8
Bradford	-2.44 (1.10)	0.97 (2.99)	7.88 (8.27)	-13.8 (12.9)	2.3, 2.2, 1.6, 1.1
**Pooled**	-3.12 (0.09)	0.58 (0.12)	0.10 (0.13)	-1.67 (0.48)	-
**I^2**	83%	83%	40%	75%	-
**MTPB**					
CPP	-3.05 (0.93)	-1.15 (0.81)	-3.75 (2.18)	-5.59 (5.06)	5.5, 5.5, 2.4, 5.5
Danish	-2.56 (0.08)	-0.27 (0.11)	-2.18 (0.26)	-4.63 (0.57)	15.3, 17.7, 24.3, 17.2
Norwegian	-2.47 (0.15)	-1.18 (0.32)	-2.56 (0.80)	-7.79 (1.42)	14.8, 13.8, 10.5, 13.8
CPRD	-2.16 (0.17)	-0.79 (0.24)	-2.61 (0.66)	-7.89 (1.38)	14.5, 14.7, 12.7, 14.6
South Australian BEBOLD	-2.58 (0.15)	0.11 (0.21)	-3.66 (0.53)	-3.40 (1.13)	14.7, 15.7, 15.6, 15.5
US Vital Statistics	-1.78 (0.01)	-1.02 (0.02)	-2.77 (0.07)	-7.94 (0.11)	15.6 18.6, 28.9, 18.5
SAIL Databank	-2.37 (0.24)	-0.05 (0.55)	-2.20 (1.50)	-5.89 (2.38)	13.9, 11.5, 4.9, 11.9
Bradford	-0.45 (1.82)	-24.1 (16.9)	57.9 (38.8)	-61.1 (33.5)	5.5, 2.5, 0.6, 3.0
**Pooled**	-2.46 (0.20)	-0.52 (0.22)	-2.67 (0.31)	-5.85 (0.80)	-
**I^2**	96%	91%	68%	84%	-

1. For constant, $sBMI^{-2}$, Ln(sBMI) × $sBMI^{-2}$, and Ln(sBMI) × Ln(sBMI) × $sBMI^{-2}$, respectively

Supplementary Table S14: Random effects meta-analysis: results are the terms (and their standard errors) for each dataset, the pooled result, and the weights (sBMI=scaled BMI) – parous women

	Term				
Dataset	Constant	$sBMI^{-2}$	Ln(sBMI) × $sBMI^{-2}$	Ln(sBMI) × Ln(sBMI) × $sBMI^{-2}$	Weights (%)[1]
			**Any PTB**		
CPP	-2.97 (0.11)	0.94 (0.11)	1.61 (0.37)	0.75 (0.41)	13.8, 13.7, 11.3, 14.4
Danish	-2.62 (0.07)	0.37 (0.12)	-0.57 (0.29)	-5.90 (0.58)	13.6, 13.9, 11.7, 13.9
Norwegian	-2.47 (0.11)	0.40 (0.21)	-2.21 (0.52)	-5.37 (1.06)	13.4, 13.7, 11.1, 13.5
CPRD	-2.42 (0.12)	0.55 (0.21)	0.51 (0.54)	-3.88 (0.99)	13.1, 13.3, 10.5, 13.2
South Australian BEBOLD	-2.35 (0.13)	0.48 (0.38)	1.33 (1.03)	-7.05 (1.44)	13.3, 13.4, 10.4, 13.4
US Vital Statistics	-2.12 (0.007)	0.22 (0.01)	-0.06 (0.04)	-5.39 (0.07)	13.9, 14.5, 30.3, 14.5
SAIL Databank	-2.76 (0.15)	0.56 (0.35)	1.92 (0.96)	-5.03 (1.50)	12.9, 12.9, 9.7, 12.8
Bradford	-3.30 (0.74)	3.66 (1.22)	5.88 (3.26)	-6.82 (6.61)	5.9, 4.7, 5.0, 4.2
**Pooled**	-2.57 (0.19)	0.73 (0.19)	-0.03 (0.09)	-4.01 (1.13)	-
I^2	97%	94%	25%	97%	-
			**SPTB**		
CPP	-3.34 (0.12)	1.10 (0.12)	1.77 (0.39)	0.78 (0.43)	13.5, 13.7, 13.0, 13.7
Danish	-3.97 (0.12)	1.13 (0.18)	1.17 (0.45)	-4.67 (0.92)	13.6, 14.0, 13.4, 13.0
Norwegian	-3.00 (0.19)	-0.008 (0.55)	1.20 (1.34)	-8.47 (2.10)	13.6, 13.9, 13.4, 13.0
CPRD	-3.59 (0.14)	1.25 (0.17)	1.70 (0.43)	-1.03 (0.87)	12.8, 13.1, 12.9, 12.7
South Australian BEBOLD	-3.70 (0.18)	1.26 (0.52)	2.84 (1.41)	-3.81 (2.03)	13.0, 13.1, 13.1, 12.7
US Vital Statistics	-3.80 (0.01)	1.24 (0.02)	1.69 (0.04)	-0.79 (0.08)	17.0, 16.2, 16.5, 14.9
SAIL Databank	-3.98 (0.25)	0.83 (0.68)	5.15 (1.80)	-4.61 (2.66)	12.1, 12.1, 12.4, 12.5
Bradford	-2.26 (1.22)	-2.36 (4.59)	23.2 (11.9)	-29.6 (15.0)	4.4, 3.8, 5.3, 7.6
**Pooled**	-3.82 (0.10)	1.35 (0.14)	1.64 (0.33)	-1.48 (0.81)	-
I^2	84%	83%	79%	92%	-
			**MTPB**		
CPP	-4.03 (0.59)	-0.90 (1.40)	2.73 (3.30)	-1.36 (5.60)	11.7, 9.6, 9.0, 11.9
Danish	-2.95 (0.09)	-0.09 (0.13)	-1.63 (0.33)	-5.94 (0.67)	13.5, 15.4, 15.7, 13.2
Norwegian	-3.13 (0.11)	-0.16 (0.15)	-3.87 (0.34)	-4.78 (0.75)	13.4, 15.0, 15.5, 13.1
CPRD	-2.66 (0.18)	-0.40 (0.41)	-0.49 (1.10)	-6.94 (1.77)	13.1, 12.4, 12.6, 12.8
South Australian BEBOLD	-2.54 (0.16)	-0.12 (0.44)	-0.50 (1.19)	-9.05 (1.69)	13.2, 12.5, 12.9, 12.8
US Vital Statistics	-2.19 (0.009)	-0.62 (0.02)	-1.47 (0.07)	-7.85 (0.10)	13.9, 16.6, 17.4, 15.5
SAIL Databank	-3.11 (0.18)	0.04 (0.38)	-0.16 (1.04)	-4.84 (1.76)	13.1, 12.1, 12.5, 12.8
Bradford	-5.08 (0.96)	4.54 (1.24)	-1.03 (3.54)	5.05 (8.61)	8.1, 6.2, 4.4, 8.1
**Pooled**	-2.95 (0.22)	0.002 (0.21)	-1.39 (0.52)	-5.83 (0.69)	-
I^2	97%	88%	90%	83%	-

1. For constant, $sBMI^{-2}$, Ln(sBMI) × $sBMI^{-2}$, and Ln(sBMI) × Ln(sBMI) × $sBMI^{-2}$, respectively

Note: between dataset heterogeneity

The I^2 statistics are generally high. This likely reflects a number of features: (i) the overall prevalence of PTB varied across datasets; (ii) the exact nature of the curves in terms of location of the minimum point (if any) and the extent to which risk increased at low and/or high BMIs; (iii) since the I^2 statistic is the fraction of the overall variance that is due to between study dataset variance, the fact that several of the within-dataset estimates were very precise as a result of the large sample sizes would impact on the I^2 estimates. Bearing these factors in mind and because the relative consistency in terms of the overall shape of the risk curves with BMI, we deemed it appropriate to meta-analyse the results despite this heterogeneity.

Secondary analyses

Table S15: Adjusted odds ratios (95% CI) of any preterm, spontaneous preterm and medically indicated preterm birth by BMI category – nulliparous women

BMI category	CPP, USA	Danish linked data	Norwegian birth registry	CPRD, UK	South Australian BEBOLD	US Vital Statistics[2]	SAIL Databank, UK	Bradford, UK
**Any PTB**								
<18.5	1.23 (1.07, 1.41)	1.19 (1.12, 1.27)	1.23 (1.13, 1.35)	1.21 (1.06, 1.37)	1.36 (1.16, 1.59)	1.208 (1.195, 1.221)	1.32 (1.04, 1.69)	3.28 (1.34, 8.04)
18.5-24.9	1.00	1.00	1.00	1.00	1.00	1.00	1.00	1.00
25-29.9	1.01 (0.86, 1.19)	1.03 (0.99, 1.07)	1.15 (1.09, 1.20)	1.09 (1.02, 1.17)	1.03 (0.96, 1.12)	1.108 (1.102, 1.114)	1.01 (0.92, 1.12)	0.70 (0.43, 1.15)
30-34.9	0.76 (0.53, 1.07)	1.14 (1.08, 1.20)	1.29 (1.20, 1.37)	1.23 (1.12, 1.34)	1.22 (1.10, 1.34)	1.315 (1.306, 1.323)	1.03 (0.90, 1.17)	0.67 (0.39, 1.14)
35-39.9	0.63 (0.32, 1.25)[1]	1.19 (1.10, 1.30)	1.38 (1.24, 1.54)	1.32 (1.15, 1.51)	1.38 (1.21, 1.58)	1.491 (1.479, 1.504)	1.26 (1.07, 1.48)	0.82 (0.42, 1.61)
40+		1.11 (0.98, 1.25)	1.32 (1.10, 1.60)	1.21 (1.00, 1.47)	1.11 (0.92, 1.33)	1.715 (1.700, 1.731)	1.13 (0.91, 1.41)	1.48 (0.73, 3.02)
**SPTB**								
<18.5	1.25 (1.08, 1.45)	1.24 (1.13, 1.35)	1.30 (1.17, 1.44)	1.16 (0.99, 1.35)	1.44 (1.18, 1.77)	1.261 (1.244, 1.279)	1.37 (0.99, 1.89)	4.15 (1.57, 10.98)
18.5-24.9	1.00	1.00	1.00	1.00	1.00	1.00	1.00	1.00
25-29.9	0.98 (0.83, 1.17)	0.93 (0.89, 0.98)	1.03 (0.97, 1.09)	1.00 (0.91, 1.09)	0.92 (0.83, 1.02)	0.983 (0.976, 0.991)	0.97 (0.84, 1.11)	0.66 (0.36, 1.20)
30-34.9	0.64 (0.43, 0.94)	0.94 (0.87, 1.01)	1.07 (0.98, 1.16)	0.94 (0.83, 1.07)	1.02 (0.89, 1.16)	1.054 (1.044, 1.064)	0.85 (0.71, 1.02)	0.51 (0.25, 1.03)
35-39.9	0.64 (0.31, 1.31)[1]	0.86 (0.75, 0.98)	1.02 (0.88, 1.19)	0.96 (0.79, 1.16)	0.97 (0.80, 1.18)	1.089 (1.076, 1.102)	0.97 (0.76, 1.23)	0.63 (0.25, 1.57)
40+		0.77 (0.63, 0.94)	1.14 (0.89, 1.46)	0.99 (0.76 , 1.28)	0.90 (0.71, 1.15)	1.104 (1.089, 1.120)	0.70 (0.49, 1.00)	1.19 (0.48, 2.94)
**MPTB**								
<18.5	0.96 (0.54, 1.73)	1.15 (1.05, 1.26)	1.13 (0.97, 1.31)	1.31 (1.07, 1.60)	1.27 (1.01, 1.59)	1.129 (1.111, 1.148)	1.27 (0.89, 1.80)	1.21 (0.16, 9.30)
18.5-24.9	1.00	1.00	1.00	1.00	1.00	1.00	1.00	1.00
25-29.9	1.11 (0.61, 2.00)	1.13 (1.08, 1.19)	1.37 (1.27, 1.47)	1.26 (1.13, 1.40)	1.19 (1.07, 1.32)	1.248 (1.239, 1.257)	1.06 (0.91, 1.22)	0.82 (0.38, 1.75)
30-34.9	2.26 (1.01, 5.02)	1.35 (1.26, 1.44)	1.67 (1.51, 1.84)	1.74 (1.52, 1.99)	1.47 (1.28, 1.68)	1.609 (1.595, 1.623)	1.22 (1.03, 1.45)	1.02 (0.47, 2.20)
35-39.9	0.95 (0.13, 7.05)[1]	1.54 (1.40, 1.72)	2.05 (1.76, 2.38)	2.02 (1.68, 2.44)	1.92 (1.61, 2.29)	1.954 (1.933, 1.974)	1.57 (1.27, 1.93)	1.21 (0.44, 3.36)
40+		1.44 (1.23, 1.68)	1.72 (1.30, 2.27)	1.62 (1.23, 2.14)	1.33 (1.04, 1.71)	2.404 (2.377, 2.432)	1.63 (1.25, 2.13)	1.90 (0.64, 5.68)

1. BMI ≥35 kg/m^2 as too few individuals with BMI>40 in this dataset
2. These ORs given to 3 decimal places to distinguish limits from point estimate (not possible with 2dp)

Table S16: Adjusted odds ratios (95% CI) of any preterm, spontaneous preterm and medically indicated preterm birth by BMI category – parous women

BMI category	CPP, USA	Danish linked data	Norwegian birth registry	CPRD, UK	South Australian BEBOLD	US Vital Statistics[2]	SAIL Databank, UK	Bradford, UK
**Any PTB**								
<18.5	1.23 (1.11, 1.37)	1.52 (1.42, 1.64)	1.45 (1.32, 1.59)	1.54 (1.34, 1.77)	1.81 (1.57, 2.10)	1.467 (1.452, 1.481)	1.64 (1.35, 1.99)	2.87 (1.27, 6.52)
18.5-24.9	1.00	1.00	1.00	1.00	1.00	1.00	1.00	1.00
25-29.9	0.79 (0.73, 0.86)	1.02 (0.98, 1.06)	1.07 (1.03, 1.12)	0.92 (0.85, 0.98)	0.87 (0.82, 0.94)	0.982 (0.978, 0.986)	0.88 (0.81, 0.96)	0.42 (0.28, 0.64)
30-34.9	0.83 (0.73, 0.95)	1.08 (1.02, 1.15)	1.27 (1.19, 1.35)	0.96 (0.88, 1.06)	1.01 (0.94, 1.10)	1.070 (1.064, 1.075)	0.85 (0.77, 0.94)	0.34 (0.21, 0.56)
35-39.9	0.70 (0.57, 0.96)[1]	1.18 (1.09, 1.29)	1.45 (1.32, 1.60)	0.97 (0.85, 1.10)	1.14 (1.03, 1.27)	1.147 (1.139, 1.154)	0.90 (0.78, 1.02)	0.43 (0.23, 0.81)
40+		1.40 (1.24, 1.56)	1.33 (1.11, 1.58)	1.06 (0.90, 1.25)	1.03 (0.90, 1.17)	1.292 (1.283, 1.301)	0.97 (0.82, 1.14)	0.46 (0.22, 0.97)
**SPTB**								
<18.5	1.28 (1.14, 1.43)	1.68 (1.52, 1.86)	1.59 (1.41, 1.78)	1.61 (1.36, 1.91)	1.93 (1.61, 2.32)	1.609 (1.589, 1.629)	1.84 (1.43, 2.36)	3.24 (1.22, 8.60)
18.5-24.9	1.00	1.00	1.00	1.00	1.00	1.00	1.00	1.00
25-29.9	0.79 (0.72, 0.86)	0.85 (0.80, 0.91)	0.97 (0.92, 1.03)	0.81 (0.73, 0.89)	0.75 (0.69, 0.83)	0.834 (0.828, 0.839)	0.77 (0.68, 0.87)	0.31 (0.16, 0.58)
30-34.9	0.83 (0.72, 0.96)	0.83 (0.76, 0.91)	1.03 (0.95, 1.13)	0.74 (0.65, 0.84)	0.79 (0.71, 0.89)	0.794 (0.788, 0.801)	0.63 (0.54, 0.74)	0.29 (0.14, 0.59)
35-39.9	0.63 (0.50, 0.79)[1]	0.86 (0.74, 1.00)	1.21 (1.06, 1.38)	0.73 (0.61, 0.87)	0.77 (0.65, 0.90)	0.740 (0.732, 0.747)	0.62 (0.50, 0.77)	0.68 (0.32, 1.43)
40+		0.97 (0.79, 1.19)	1.11 (0.87, 1.41)	0.74 (0.58, 0.94)	0.65 (0.53, 0.80)	0.671 (0.663, 0.680)	0.65 (0.50, 0.84)	0.28 (0.08, 1.00)
**MPTB**								
<18.5	0.94 (0.67, 1.32)	1.40 (1.27, 1.54)	1.22 (1.05, 1.43)	1.41 (1.12, 1.77)	1.66 (1.32, 2.09)	1.275 (1.256, 1.294)	1.43 (1.06, 1.91)	1.90 (0.56, 6.50)
18.5-24.9	1.00	1.00	1.00	1.00	1.00	1.00	1.00	1.00
25-29.9	0.73 (0.57, 0.93)	1.13 (1.07, 1.19)	1.22 (1.14, 1.30)	1.08 (0.97, 1.20)	1.02 (0.93, 1.13)	1.139 (1.132, 1.146)	1.00 (0.89, 1.13)	0.59 (0.34, 1.03)
30-34.9	0.97 (0.68, 1.38)	1.26 (1.18, 1.35)	1.58 (1.45, 1.73)	1.30 (1.14, 1.47)	1.31 (1.18, 1.46)	1.365 (1.355, 1.374)	1.07 (0.94, 1.23)	0.46 (0.24, 0.86)
35-39.9	1.12 (0.70, 1.78)[1]	1.40 (1.27, 1.55)	1.79 (1.57, 2.04)	1.33 (1.12, 1.58)	1.60 (1.40, 1.83)	1.588 (1.575, 1.601)	1.17 (0.99, 1.38)	0.26 (0.09, 0.74)
40+		1.68 (1.47, 1.92)	1.60 (1.26, 2.03)	1.53 (1.24, 1.89)	1.51 (1.28, 1.77)	1.968 (1.951, 1.985)	1.27 (1.04, 1.54)	0.76 (0.33, 1.77)

1. BMI ≥35 kg/m^2 as too few individuals with BMI>40 in this dataset
2. These ORs given to 3 decimal places to distinguish limits from point estimate (not possible with 2dp)

Supplementary Table S17: Adjusted odds ratios (95% CI) for any very (<32 completed weeks) preterm, spontaneous very preterm and medically indicated very preterm birth by BMI category – nulliparous women

BMI category	CPP, USA	Danish linked data	Norwegian birth registry	CPRD, UK	South Australian BEBOLD	US vital statistics	SAIL Databank, UK	Bradford, UK[3]
**Any very preterm birth**								
<18.5	1.03 (0.79, 1.35)	1.07 (0.91, 1.25)	1.17 (0.94, 1.47)	1.16 (0.94, 1.43)	1.06 (0.66, 1.71)	1.14 (1.11, 1.17)	1.92 (1.18, 3.12)	[3]
18.5-24.9	1.00	1.00	1.00	1.00	1.00	1.00	1.00	
25-29.9	1.14 (0.86, 1.51)	1.09 (1.00, 1.18)	1.31 (1.17, 1.46)	1.14 (1.02, 1.28)	1.04 (0.85, 1.28)	1.20 (1.19, 1.22)	1.01 (0.79, 1.28)	
30-34.9	0.99 (0.56, 1.76)	1.35 (1.21, 1.52)	1.57 (1.35, 1.82)	1.27 (1.09, 1.48)	1.37 (1.07, 1.76)	1.57 (1.54, 1.59)	1.29 (0.97, 1.71)	
35-39.9	1.21 (0.48, 3.08)[1]	1.37 (1.14, 1.64)	2.00 (1.60, 2.50)	1.26 (1.00, 1.59)	1.76 (1.29, 2.41)	1.84 (1.81, 1.87)	1.12 (0.75, 1.66)	
40+		1.51 (1.16, 1.95)	2.22 (1.52, 3.25)	1.04 (0.73, 1.47)	1.55 (1.07, 2.25)	2.06 (2.02, 2.09)	1.12 (0.67, 1.88)	
**Spontaneous very preterm birth**								
<18.5	1.04 (0.78, 1.39)	1.16 (0.92, 1.47)	1.08 (0.82, 1.43)	1.26 (0.99, 1.61)	1.41 (0.75, 2.65)	1.17 (1.13, 1.21)	2.12 (1.17, 3.84)	[3]
18.5-24.9	1.00	1.00	1.00	1.00	1.00	1.00	1.00	
25-29.9	1.12 (0.82, 1.53)	1.03 (0.90, 1.18)	1.16 (1.01, 1.33)	1.08 (0.94, 1.25)	1.01 (0.75, 1.35)	1.14 (1.12, 1.16)	0.79 (0.57, 1.11)	
30-34.9	1.03 (0.60, 1.77)[2]	1.20 (0.99, 1.45)	1.45 (1.20, 1.76)	0.99 (0.81, 1.22)	1.37 (0.96, 1.97)	1.46 (1.43, 1.49)	1.09 (0.74, 1.59)	
35-39.9		1.31 (0.98, 1.75)	1.83 (1.38, 2.42)	0.84 (0.59, 1.18)	1.82 (1.17, 2.81)	1.72 (1.67, 1.76)	0.73 (0.40, 1.34)	
40+		1.30 (0.86, 1.98)	2.39 (1.53, 3.73)	0.99 (0.64, 1.54)	1.83 (1.12, 2.99)	1.87 (1.82, 1.92)	0.69 (0.30, 1.58)	
**Medically indicated very preterm birth**								
<18.5	1.17 (0.40, 3.43)	1.00 (0.81, 1.24)	1.36 (0.95, 1.95)	0.96 (0.64, 1.42)	0.81 (0.39, 1.66)	1.13 (1.09, 1.17)	1.55 (0.67, 3.57)	[3]
18.5-24.9	1.00	1.00	1.00	1.00	1.00	1.00	1.00	
25-29.9	0.96 (0.28, 3.29)	1.12 (1.01, 1.25)	1.61 (1.35, 1.92)	1.24 (1.03, 1.49)	1.09 (0.83, 1.42)	1.27 (1.25, 1.29)	1.27 (0.89, 1.81)	
30-34.9	0.87 (0.11, 6.73)[2]	1.45 (1.26, 1.67)	1.78 (1.39, 2.27)	1.75 (1.39, 2.19)	1.37 (0.99, 1.90)	1.67 (1.64, 1.70)	1.57 (1.04, 2.38)	
35-39.9		1.41 (1.11, 1.77)	2.31 (1.61, 3.32)	2.09 (1.53, 2.85)	1.71 (1.13, 2.59)	1.98 (1.94, 2.02)	1.69 (1.00, 2.85)	
40+		1.62 (1.18, 2.22)	1.84 (0.90, 3.73)	1.07 (0.60, 1.91)	1.31 (0.76, 2.25)	2.23 (2.18, 2.29)	1.78 (0.92, 3.47)	

1. BMI ≥35 kg/m^2 as too few individuals with BMI>40 in this dataset
2. BMI ≥30 kg/m^2 as numbers with outcomes too small in higher BMI groups
3. Numbers too small to carry out this analysis

Supplementary Table S18: Adjusted odds ratios (95% CI) for any very (<32 completed weeks) preterm, spontaneous very preterm and medically indicated very preterm birth by BMI category – parous women

BMI category	CPP, USA	Danish linked data	Norwegian birth registry	CPRD, UK	South Australian BEBOLD	US vital statistics	SAIL Databank, UK	Bradford, UK[2]
**Any very preterm birth**								
<18.5	1.27 (1.05, 1.53)	1.58 (1.32, 1.88)	1.55 (1.22, 1.96)	1.31 (1.03, 1.67)	1.79 (1.23, 2.60)	1.47 (1.44, 1.51)	1.33 (0.81, 2.17)	[2]
18.5-24.9	1.00	1.00	1.00	1.00	1.00	1.00	1.00	
25-29.9	0.81 (0.70, 0.95)	1.16 (1.05, 1.27)	1.14 (1.01, 1.27)	0.87 (0.77, 0.99)	0.79 (0.65, 0.95)	0.99 (0.98, 1.00)	0.87 (0.71, 1.08)	
30-34.9	0.87 (0.68, 1.11)	1.17 (1.03, 1.34)	1.38 (1.19, 1.60)	0.94 (0.81, 1.10)	0.94 (0.75, 1.17)	1.08 (1.07, 1.10)	0.87 (0.67, 1.11)	
35-39.9	0.72 (0.50, 1.05)[1]	1.14 (0.93, 1.41)	1.24 (0.97, 1.59)	1.07 (0.87, 1.30)	1.02 (0.76, 1.37)	1.14 (1.12, 1.16)	0.91 (0.67, 1.24)	
40+		1.63 (1.26, 2.09)	1.82 (1.26, 2.63)	0.97 (0.73, 1.29)	0.72 (0.49, 1.05)	1.19 (1.17, 1.21)	1.00 (0.69, 1.46)	
**Spontaneous very preterm birth**								
<18.5	1.24 (1.00, 1.53)	1.59 (1.19, 2.11)	1.81 (1.35, 2.41)	1.43 (1.07, 1.90)	2.22 (1.41, 3.50)	1.59 (1.54, 1.65)	1.56 (0.83, 2.91)	[2]
18.5-24.9	1.00	1.00	1.00	1.00	1.00	1.00	1.00	
25-29.9	0.78 (0.66, 0.93)	0.99 (0.83, 1.16)	1.13 (0.97, 1.30)	0.79 (0.68, 0.92)	0.60 (0.46, 0.80)	0.87 (0.85, 0.88)	0.77 (0.57, 1.03)	
30-34.9	0.87 (0.66, 1.14)	0.87 (0.68, 1.12)	1.30 (1.06, 1.59)	0.74 (0.60, 0.91)	0.82 (0.60, 1.11)	0.88 (0.86, 0.90)	0.84 (0.60, 1.19)	
35-39.9	0.65 (0.41, 1.01)[1]	0.93 (0.64, 1.38)	1.15 (0.83, 1.61)	0.71 (0.53, 0.95)	0.74 (0.47, 1.14)	0.87 (0.84, 0.89)	0.78 (0.50, 1.22)	
40+		1.25 (0.79, 1.97)	1.88 (1.18, 3.00)	0.68 (0.45, 1.02)	0.47 (0.26, 0.83)	0.82 (0.79, 0.84)	0.90 (0.53, 1.55)	
**Medically indicated very preterm birth**								
<18.5	1.51 (0.77, 2.96)	1.57 (1.26, 1.96)	1.16 (0.78, 1.74)	1.08 (0.70, 1.69)	1.30 (0.68, 2.51)	1.40 (1.35, 1.45)	1.09 (0.50, 2.37)	[2]
18.5-24.9	1.00	1.00	1.00	1.00	1.00	1.00	1.00	
25-29.9	0.83 (0.49, 1.42)	1.25 (1.11, 1.41)	1.15 (0.96, 1.37)	1.03 (0.85, 1.24)	1.00 (0.78, 1.28)	1.08 (1.06, 1.09)	1.00 (0.74, 1.34)	
30-34.9	1.04 (0.49, 2.20)	1.34 (1.15, 1.57)	1.50 (1.20, 1.87)	1.29 (1.03, 1.62)	1.07 (0.79, 1.44)	1.22 (1.20, 1.25)	0.90 (0.63, 1.29)	
35-39.9	0.81 (0.25, 2.67)[1]	1.26 (0.98, 1.62)	1.37 (0.95, 1.98)	1.71 (1.30, 2.26)	1.34 (0.92, 1.95)	1.33 (1.30, 1.36)	1.02 (0.66, 1.58)	
40+		1.84 (1.37, 2.47)	1.72 (0.96, 3.08)	1.54 (1.06, 2.24)	1.04 (0.64, 1.71)	1.44 (1.41, 1.48)	1.12 (0.67, 1.86)	

1. BMI ≥35 kg/m^2 as too few individuals with BMI>40 in this dataset
2. Numbers too small to carry out this analysis

Sensitivity analyses

Supplementary Table S19: Adjusted odds ratios (95% CI) for any preterm, spontaneous preterm and medically indicated preterm birth by BMI category: inverse probability weighted[1] – nulliparous women

**SPTB**								
BMI category	CPP, USA	Danish linked data	Norwegian birth registry	CPRD, UK	South Australian BEBOLD	US vital statistics	SAIL Databank, UK	Bradford, UK
<18.5	1.25 (1.08, 1.45)	1.24 (1.14, 1.36)	1.30 (1.17, 1.44)	1.15 (0.98, 1.35)	1.43 (1.16, 1.76)	1.26 (1.24, 1.28)	1.36 (0.98, 1.88)	4.21 (1.58, 11.18)
18.5-24.9	1.00	1.00	1.00	1.00	1.00	1.00	1.00	1.00
25-29.9	0.98 (0.83, 1.17)	0.93 (0.89, 0.98)	1.02 (0.96, 1.08)	0.99 (0.91, 1.09)	0.92 (0.82, 1.02)	0.98 (0.98, 0.99)	0.97 (0.84, 1.11)	0.67 (0.37, 1.24)
30-34.9	0.64 (0.43, 0.94)	0.94 (0.87, 1.02)	1.06 (0.97, 1.15)	0.96 (0.84, 1.08)	0.98 (0.85, 1.14)	1.05 (1.04, 1.06)	0.84 (0.70, 1.01)	0.50 (0.24, 1.03)
35-39.9	0.64 (0.30, 1.36)[2]	0.87 (0.76, 0.99)	1.01 (0.87, 1.18)	0.98 (0.81, 1.19)	0.98 (0.79, 1.22)	1.09 (1.07, 1.10)	0.95 (0.74, 1.21)	0.73 (0.29, 1.87)
40+		0.74 (0.60, 0.91)	1.18 (0.93, 1.51)	1.01 (0.78, 1.31)	0.88 (0.67, 1.15)	1.08 (1.06, 1.10)	0.69 (0.48, 0.99)	1.32 (0.54, 3.20)
**MPTB**								
<18.5	0.99 (0.55, 1.77)	1.15 (1.05, 1.26)	1.16 (1.00, 1.34)	1.31 (1.07, 1.61)	1.35 (1.08, 1.70)	1.13 (1.11, 1.15)	1.28 (0.90, 1.82)	1.22 (0.16, 9.40)
18.5-24.9	1.00	1.00	1.00	1.00	1.00	1.00	1.00	1.00
25-29.9	1.07 (0.59, 1.96)	1.12 (1.07, 1.18)	1.37 (1.27, 1.47)	1.26 (1.13, 1.40)	1.20 (1.08, 1.34)	1.24 (1.23, 1.25)	1.06 (0.92, 1.22)	0.81 (0.38, 1.73)
30-34.9	2.21 (0.98, 4.99)	1.35 (1.26, 1.44)	1.61 (1.46, 1.79)	1.74 (1.52, 1.98)	1.47 (1.28, 1.69)	1.59 (1.58, 1.61)	1.21 (1.02, 1.44)	1.03 (0.48, 2.21)
35-39.9	0.95 (0.13, 6.69)[2]	1.53 (1.38, 1.70)	2.01 (1.73, 2.34)	2.02 (1.68, 2.44)	1.90 (1.59, 2.28)	1.93 (1.91, 1.95)	1.54 (1.25, 1.91)	1.21 (0.44, 3.37)
40+		1.42 (1.21, 1.67)	1.76 (1.33, 2.32)	1.62 (1.23, 2.14)	1.27 (0.98, 1.65)	2.36 (2.34, 2.39)	1.63 (1.25, 2.12)	1.92 (0.63, 5.81)

1. Weighting SPTB by the inverse of one minus the probability of being a medically indicated preterm birth and weighting medically indicated preterm birth by the inverse of one minus the probability of being a SPTB
2. BMI ≥35 kg/m^2 as too few individuals with BMI of 40 or higher in this dataset

Supplementary Table S20: Adjusted odds ratios (95% CI) for any preterm, spontaneous preterm and medically indicated preterm birth by BMI category: inverse probability weighted[1] – parous women

BMI category	CPP, USA	Danish linked data	Norwegian birth registry	CPRD, UK	South Australian BEBOLD	US vital statistics	SAIL Databank, UK	Bradford, UK
**SPTB**								
<18.5	1.28 (1.14, 1.43)	1.68 (1.52, 1.86)	1.58 (1.40, 1.77)	1.61 (1.36, 1.91)	2.03 (1.69, 2.44)	1.61 (1.59, 1.63)	1.86 (1.45, 2.40)	3.21 (1.21, 8.52)
18.5-24.9	1.00	1.00	1.00	1.00	1.00	1.00	1.00	1.00
25-29.9	0.79 (0.72, 0.86)	0.85 (0.80, 0.91)	0.97 (0.91, 1.03)	0.80 (0.73, 0.88)	0.77 (0.70, 0.85)	0.83 (0.83, 0.84)	0.77 (0.68, 0.87)	0.32 (0.17, 0.62)
30-34.9	0.83 (0.72, 0.96)	0.83 (0.76, 0.91)	1.03 (0.94, 1.12)	0.74 (0.65, 0.84)	0.85 (0.75, 0.95)	0.79 (0.79, 0.80)	0.65 (0.55, 0.76)	0.30 (0.15, 0.62)
35-39.9	0.63 (0.49, 0.79)[2]	0.84 (0.72, 0.98)	1.20 (1.05, 1.37)	0.73 (0.61, 0.88)	0.76 (0.63, 0.91)	0.74 (0.73, 0.74)	0.64 (0.52, 0.79)	0.69 (0.32, 1.45)
40+		0.95 (0.77, 1.17)	1.09 (0.85, 1.39)	0.74 (0.58, 0.94)	0.67 (0.54, 0.83)	0.66 (0.65, 0.67)	0.63 (0.47, 0.83)	0.27 (0.07, 0.99)
**MPTB**								
<18.5	0.90 (0.64, 1.27)	1.40 (1.27, 1.54)	1.23 (1.05, 1.43)	1.37 (1.09, 1.73)	1.65 (1.30, 2.10)	1.28 (1.27, 1.30)	1.45 (1.08, 1.95)	2.02 (0.59, 6.85)
18.5-24.9	1.00	1.00	1.00	1.00	1.00	1.00	1.00	1.00
25-29.9	0.74 (0.57, 0.94)	1.13 (1.07, 1.18)	1.21 (1.13, 1.30)	1.07 (0.96, 1.19)	1.02 (0.92, 1.12)	1.13 (1.12, 1.14)	1.01 (0.89, 1.13)	0.58 (0.33, 1.01)
30-34.9	1.01 (0.71, 1.44)	1.26 (1.18, 1.35)	1.56 (1.42, 1.70)	1.27 (1.11, 1.44)	1.31 (1.17, 1.46)	1.35 (1.34, 1.36)	1.08 (0.94, 1.23)	0.45 (0.24, 0.84)
35-39.9	1.13 (0.71, 1.81)[2]	1.40 (1.27, 1.55)	1.76 (1.54, 2.01)	1.31 (1.10, 1.56)	1.56 (1.36, 1.80)	1.56 (1.55, 1.58)	1.18 (0.99, 1.39)	0.25 (0.09, 0.72)
40+		1.67 (1.46, 1.91)	1.56 (1.23, 2.00)	1.52 (1.22, 1.87)	1.49 (1.26, 1.75)	1.93 (1.91, 1.95)	1.24 (1.01, 1.52)	0.76 (0.33, 1.78)

1. Weighting SPTB by the inverse of one minus the probability of being a medically indicated preterm birth and weighting medically indicated preterm birth by the inverse of one minus the probability of being a SPTB
2. BMI ≥35 kg/m^2 as too few individuals with BMI of 40 or higher in this dataset

Supplementary Table S21: Adjusted odds ratios (95% CI) for any preterm, spontaneous preterm and medically indicated preterm birth by BMI category excluding stillbirths – nulliparous women

Any PTB								
BMI category	CPP, USA	Danish linked data	Norwegian birth registry	CPRD, UK	South Australian BEBOLD	US vital statistics	SAIL Databank, UK	Bradford, UK
<18.5	1.21 (1.05, 1.40)	1.20 (1.12, 1.28)	1.24 (1.13, 1.35)	1.21 (1.07, 1.38)	1.33 (1.14, 1.56)	1.21 (1.20, 1.22)	1.27 (0.99, 1.64)	3.41 (1.37, 8.46)
18.5-24.9	1.00	1.00	1.00	1.00	1.00	1.00	1.00	1.00
25-29.9	0.96 (0.81, 1.14)	1.03 (0.99, 1.07)	1.14 (1.09, 1.19)	1.09 (1.02, 1.17)	1.04 (0.96, 1.13)	1.11 (1.10, 1.11)	0.99 (0.90, 1.10)	0.74 (0.45, 1.21)
30-34.9	0.71 (0.49, 1.04)	1.13 (1.07, 1.19)	1.29 (1.20, 1.38)	1.23 (1.12, 1.35)	1.23 (1.11, 1.36)	1.31 (1.30, 1.32)	1.09 (0.88, 1.14)	0.61 (0.35, 1.08)
35-39.9	0.63 (0.31, 1.29)[1]	1.17 (1.08, 1.28)	1.36 (1.22, 1.52)	1.32 (1.15, 1.52)	1.38 (1.20, 1.58)	1.49 (1.48, 1.50)	1.23 (1.04, 1.45)	0.80 (0.39, 1.63)
40+		1.08 (0.95, 1.23)	1.30 (1.07, 1.57)	1.22 (1.00, 1.48)	1.12 (0.93, 1.34)	1.71 (1.70, 1.73)	1.09 (0.88, 1.37)	1.44 (0.66, 3.13)
**SPTB**								
<18.5	1.24 (1.07, 1.43)	1.24 (1.13, 1.35)	1.30 (1.17, 1.45)	1.15 (0.99, 1.35)	1.45 (1.19, 1.78)	1.26 (1.24, 1.28)	1.36 (0.98, 1.88)	4.13 (1.55, 11.05)
18.5-24.9	1.00	1.00	1.00	1.00	1.00	1.00	1.00	1.00
25-29.9	0.94 (0.79, 1.13)	0.93 (0.88, 0.98)	1.01 (0.96, 1.07)	1.00 (0.92, 1.09)	0.92 (0.83, 1.02)	0.98 (0.98, 0.99)	0.97 (0.85, 1.12)	0.66 (0.36, 1.19)
30-34.9	0.63 (0.42, 0.95)	0.93 (0.86, 1.01)	1.06 (0.97, 1.15)	0.95 (0.84, 1.08)	1.02 (0.89, 1.17)	1.05 (1.04, 1.06)	0.85 (0.70, 1.02)	0.51 (0.25, 1.03)
35-39.9	0.61 (0.29, 1.31)[1]	0.85 (0.74, 0.97)	1.01 (0.87, 1.17)	0.96 (0.79, 1.17)	0.97 (0.80, 1.18)	1.09 (1.08, 1.10)	0.97 (0.76, 1.23)	0.63 (0.25, 1.58)
40+		0.76 (0.62, 0.93)	1.10 (0.86, 1.42)	0.99 (0.76, 1.28)	0.90 (0.70, 1.16)	1.11 (1.09, 1.12)	0.67 (0.46, 0.97)	1.20 (0.47, 3.09)
**MPTB**								
<18.5	0.82 (0.43, 1.59)	1.16 (1.05, 1.27)	1.12 (0.97, 1.31)	1.33 (1.09, 1.64)	1.20 (0.94, 1.52)	1.13 (1.11, 1.15)	1.17 (0.81, 1.70)	1.34 (0.17, 10.86)
18.5-24.9	1.00	1.00	1.00	1.00	1.00	1.00	1.00	1.00
25-29.9	1.15 (0.62, 2.12)	1.13 (1.07, 1.18)	1.37 (1.28, 1.48)	1.27 (1.14, 1.42)	1.21 (1.08, 1.35)	1.25 (1.24, 1.26)	1.01 (0.87, 1.17)	0.94 (0.42, 2.12)
30-34.9	2.23 (0.94, 5.29)	1.33 (1.24, 1.43)	1.69 (1.53, 1.87)	1.76 (1.54, 2.02)	1.49 (1.30, 1.71)	1.61 (1.60, 1.62)	1.17 (0.98, 1.40)	0.87 (0.36, 2.13)
35-39.9	1.10 (0.15, 8.23)[1]	1.52 (1.37, 1.70)	2.02 (1.73, 2.35)	2.07 (1.71, 2.50)	1.93 (1.62, 2.31)	1.96 (1.93, 1.98)	1.50 (1.21, 1.87)	1.16 (0.39, 3.49)
40+		1.40 (1.19, 1.65)	1.72 (1.30, 2.28)	1.67 (1.26, 2.20)	1.36 (1.06, 1.75)	2.41 (2.38, 2.43)	1.60 (1.21, 2.10)	1.78 (0.52, 6.02)

1. BMI ≥35 kg/m^2 as too few individuals with BMI>40 in this dataset
2. These ORs given to 3 decimal places to distinguish limits from point estimate (not possible with 2dp)

Supplementary Table S22: Adjusted odds ratios (95% CI) for any preterm, spontaneous preterm and medically indicated preterm birth by BMI category excluding stillbirths – parous women

**Any PTB**								
BMI category	CPP, USA	Danish linked data	Norwegian birth registry	CPRD, UK	South Australian BEBOLD	US vital statistics	SAIL Databank, UK	Bradford, UK
<18.5	1.22 (1.10, 1.36)	1.54 (1.43, 1.66)	1.48 (1.35, 1.63)	1.54 (1.34, 1.78)	1.87 (1.61, 2.17)	1.47 (1.46, 1.49)	1.64 (1.34, 2.00)	2.22 (0.90, 5.49)
18.5-24.9	1.00	1.00	1.00	1.00	1.00	1.00	1.00	1.00
25-29.9	0.77 (0.70, 0.84)	1.00 (0.96, 1.05)	1.08 (1.03, 1.13)	0.92 (0.85, 0.99)	0.88 (0.82, 0.94)	0.98 (0.98, 0.98)	0.86 (0.79, 0.94)	0.38 (0.24, 0.60)
30-34.9	0.83 (0.72, 0.95)	1.07 (1.02, 1.14)	1.26 (1.19, 1.35)	0.96 (0.88, 1.05)	1.02 (0.94, 1.10)	1.07 (1.06, 1.07)	0.84 (0.75, 0.93)	0.33 (0.19, 0.54)
35-39.9	0.67 (0.54, 0.83)[1]	1.17 (1.07, 1.28)	1.46 (1.32, 1.61)	0.98 (0.86, 1.11)	1.14 (1.03, 1.27)	1.14 (1.14, 1.15)	0.88 (0.77, 1.01)	0.48 (0.26, 0.88)
40+		1.36 (1.21, 1.53)	1.29 (1.08, 1.54)	1.05 (0.89, 1.24)	1.03 (0.90, 1.17)	1.29 (1.28, 1.30)	0.93 (0.79, 1.10)	0.48 (0.23, 0.99)
**SPTB**								
<18.5	1.27 (1.13, 1.42)	1.70 (1.53, 1.88)	1.61 (1.43, 1.80)	1.61 (1.35, 1.91)	1.96 (1.64, 2.36)	1.61 (1.59, 1.63)	1.85 (1.44, 2.39)	2.79 (0.98, 7.96)
18.5-24.9	1.00	1.00	1.00	1.00	1.00	1.00	1.00	1.00
25-29.9	0.78 (0.71, 0.86)	0.85 (0.79, 0.90)	0.96 (0.91, 1.02)	0.81 (0.73, 0.89)	0.76 (0.69, 0.83)	0.83 (0.83, 0.84)	0.76 (0.67, 0.86)	0.31 (0.16, 0.60)
30-34.9	0.83 (0.72, 0.96)	0.83 (0.76, 0.91)	1.02 (0.94, 1.12)	0.73 (0.65, 0.83)	0.79 (0.71, 0.89)	0.79 (0.79, 0.80)	0.64 (0.54, 0.75)	0.29 (0.14, 0.61)
35-39.9	0.63 (0.50, 0.80)[1]	0.86 (0.74, 1.00)	1.19 (1.04, 1.36)	0.73 (0.61, 0.87)	0.77 (0.66, 0.91)	0.74 (0.73, 0.75)	0.62 (0.50, 0.77)	0.72 (0.34, 1.51)
40+		0.97 (0.79, 1.18)	1.04 (0.81, 1.34)	0.74 (0.58, 0.94)	0.66 (0.54, 0.80)	0.67 (0.66, 0.68)	0.63 (0.48, 0.82)	0.28 (0.08, 0.94)
**MPTB**								
<18.5	0.95 (0.67, 1.36)	1.41 (1.28, 1.55)	1.27 (1.09, 1.48)	1.42 (1.13, 1.78)	1.74 (1.38, 2.19)	1.28 (1.26, 1.30)	1.40 (1.03, 1.90)	1.32 (0.29, 5.96)
18.5-24.9	1.00	1.00	1.00	1.00	1.00	1.00	1.00	1.00
25-29.9	0.71 (0.55, 0.93)	1.11 (1.06, 1.17)	1.24 (1.16, 1.32)	1.09 (0.98, 1.22)	1.04 (0.94, 1.14)	1.14 (1.13, 1.15)	0.98 (0.87, 1.10)	0.51 (0.28, 0.94)
30-34.9	0.89 (0.60, 1.31)	1.25 (1.17, 1.34)	1.60 (1.47, 1.75)	1.30 (1.14, 1.48)	1.33 (1.20, 1.49)	1.36 (1.35, 1.37)	1.06 (0.92, 1.21)	0.43 (0.22, 0.84)
35-39.9	1.03 (0.61, 1.72)[1]	1.39 (1.25, 1.54)	1.83 (1.60, 2.09)	1.36 (1.14, 1.61)	1.62 (1.41, 1.86)	1.59 (1.57, 1.60)	1.15 (0.97, 1.37)	0.29 (0.10, 0.84)
40+		1.64 (1.42, 1.88)	1.62 (1.27, 2.06)	1.53 (1.24, 1.90)	1.52 (1.29, 1.79)	1.97 (1.95, 1.98)	1.22 (0.99, 1.50)	0.82 (0.34, 1.96)

1. BMI ≥35 kg/m^2 as too few individuals with BMI>40 in this dataset
2. These ORs given to 3 decimal places to distinguish limits from point estimate (not possible with 2dp)

Supplementary Table S23: Adjusted odds ratios (95% CI) for any preterm, spontaneous preterm and medically indicated preterm birth by BMI category excluding post term births – nulliparous women

**Any PTB**								
BMI category	CPP, USA	Danish linked data	Norwegian birth registry	CPRD, UK	South Australian BEBOLD	US vital statistics	SAIL Databank, UK	Bradford, UK
<18.5	1.25 (1.08, 1.44)	1.17 (1.10, 1.25)	1.21 (1.11, 1.32)	1.20 (1.06, 1.36)	1.36 (1.16, 1.59)	1.21 (1.19, 1.22)	1.32 (1.04, 1.69)	3.25 (1.32, 8.06)
18.5-24.9	1.00	1.00	1.00	1.00	1.00	1.00	1.00	1.00
25-29.9	1.06 (0.90, 1.26)	1.05 (1.01, 1.08)	1.16 (1.11, 1.22)	1.10 (1.03, 1.18)	1.03 (0.96, 1.12)	1.11 (1.10, 1.11)	1.03 (0.93, 1.14)	0.71 (0.43, 1.15)
30-34.9	0.86 (0.60, 1.24)	1.16 (1.10, 1.22)	1.29 (1.21, 1.38)	1.24 (1.13, 1.36)	1.22 (1.10, 1.34)	1.31 (1.30, 1.32)	1.05 (0.92, 1.19)	0.68 (0.40, 1.17)
35-39.9	0.74 (0.37, 1.49)[1]	1.21 (1.12, 1.32)	1.39 (1.25, 1.54)	1.33 (1.16, 1.53)	1.39 (1.21, 1.58)	1.49 (1.48, 1.50)	1.27 (1.08, 1.50)	0.83 (0.42, 1.64)
40+		1.13 (1.00, 1.28)	1.30 (1.07, 1.57)	1.23 (1.01, 1.50)	1.11 (0.92, 1.32)	1.71 (1.70, 1.73)	1.14 (0.92, 1.42)	1.51 (0.72, 3.17)
**SPTB**								
<18.5	1.27 (1.10, 1.48)	1.22 (1.12, 1.33)	1.27 (1.14, 1.40)	1.15 (0.98, 1.34)	1.44 (1.18, 1.77)	1.26 (1.24, 1.28)	1.37 (0.99, 1.90)	4.12 (1.54, 11.02)
18.5-24.9	1.00	1.00	1.00	1.00	1.00	1.00	1.00	1.00
25-29.9	1.03 (0.86, 1.23)	0.95 (0.89, 1.00)	1.04 (0.98, 1.11)	1.01 (0.92, 1.10)	0.92 (0.83, 1.02)	0.98 (0.98, 0.99)	0.98 (0.85, 1.13)	0.66 (0.36, 1.21)
30-34.9	0.72 (0.48, 1.08)	0.96 (0.88, 1.03)	1.07 (0.99, 1.17)	0.95 (0.84, 1.08)	1.02 (0.89, 1.16)	1.05 (1.04, 1.06)	0.87 (0.72, 1.04)	0.51 (0.25, 1.04)
35-39.9	0.75 (0.36, 1.56)[1]	0.88 (0.77, 1.00)	1.03 (0.89, 1.19)	0.97 (0.80, 1.18)	0.98 (0.80, 1.19)	1.09 (1.07, 1.10)	0.98 (0.77, 1.24)	0.63 (0.25, 1.59)
40+		0.78 (0.64, 0.96)	1.12 (0.88, 1.43)	1.01 (0.78, 1.30)	0.90 (0.70, 1.15)	1.10 (1.09, 1.12)	0.70 (0.49, 1.01)	1.20 (0.47, 3.08)
**MPTB**								
<18.5	0.97 (0.54, 1.75)	1.13 (1.03, 1.24)	1.10 (0.95, 1.28)	1.30 (1.06, 1.59)	1.27 (1.01, 1.60)	1.13 (1.11, 1.15)	1.27 (0.89, 1.80)	1.20 (0.15, 9.55)
18.5-24.9	1.00	1.00	1.00	1.00	1.00	1.00	1.00	1.00
25-29.9	1.17 (0.65, 2.11)	1.14 (1.09, 1.20)	1.39 (1.29, 1.49)	1.28 (1.15, 1.42)	1.19 (1.07, 1.32)	1.25 (1.24, 1.26)	1.07 (0.93, 1.24)	0.82 (0.38, 1.79)
30-34.9	2.53 (1.13, 5.69)	1.37 (1.28, 1.47)	1.67 (1.51, 1.85)	1.76 (1.54, 2.01)	1.47 (1.28, 1.68)	1.61 (1.59, 1.62)	1.24 (1.05, 1.48)	1.04 (0.47, 2.30)
35-39.9	1.12 (0.15, 8.40)[1]	1.57 (1.41, 1.74)	2.05 (1.76, 2.38)	2.04 (1.69, 2.47)	1.92 (1.61, 2.29)	1.95 (1.93, 1.97)	1.59 (1.28, 1.96)	1.21 (0.44, 3.30)
40+		1.47 (1.25, 1.72)	1.68 (1.28, 2.22)	1.65 (1.25, 2.17)	1.33 (1.04, 1.70)	2.40 (2.37, 2.43)	1.63 (1.25, 2.14)	1.93 (0.65, 5.78)

1. BMI ≥35 kg/m^2 as too few individuals with BMI>40 in this dataset

Supplementary Table S24: Adjusted odds ratios (95% CI) for any preterm, spontaneous preterm and medically indicated preterm birth by BMI category excluding post term births – parous women

**Any PTB**								
BMI category	CPP, USA	Danish linked data	Norwegian birth registry	CPRD, UK	South Australian BEBOLD	US vital statistics	SAIL Databank, UK	Bradford, UK
<18.5	1.20 (1.08, 1.33)	1.50 (1.40, 1.62)	1.43 (1.30, 1.57)	1.53 (1.33, 1.76)	1.81 (1.56, 2.10)	1.47 (1.45, 1.48)	1.63 (1.34, 1.98)	2.88 (1.25, 6.61)
18.5-24.9	1.00	1.00	1.00	1.00	1.00	1.00	1.00	1.00
25-29.9	0.81 (0.74, 0.88)	1.02 (0.98, 1.07)	1.08 (1.03, 1.13)	0.92 (0.85, 0.99)	0.87 (0.82, 0.94)	0.98 (0.98, 0.99)	0.87 (0.81, 0.97)	0.42 (0.27, 0.65)
30-34.9	0.86 (0.75, 0.99)	1.09 (1.03, 1.16)	1.27 (1.19, 1.35)	0.97 (0.89, 1.06)	1.01 (0.94, 1.10)	1.07 (1.06, 1.07)	0.86 (0.77, 0.95)	0.34 (0.21, 0.56)
35-39.9	0.78 (0.63, 0.96)[1]	1.19 (1.10, 1.30)	1.45 (1.32, 1.59)	0.98 (0.86, 1.11)	1.14 (1.02, 1.27)	1.15 (1.14, 1.15)	0.90 (0.79, 1.03)	0.44 (0.23, 0.81)
40+		1.40 (1.24, 1.57)	1.32 (1.11, 1.57)	1.07 (0.91, 1.26)	1.03 (0.90, 1.17)	1.29 (1.28, 1.30)	0.97 (0.83, 1.14)	0.46 (0.22, 0.96)
**SPTB**								
<18.5	1.24 (1.11, 1.39)	1.66 (1.50, 1.84)	1.57 (1.40, 1.76)	1.60 (1.35, 1.90)	1.93 (1.61, 2.32)	1.61 (1.59, 1.63)	1.82 (1.42, 2.35)	3.24 (1.21, 8.68)
18.5-24.9	1.00	1.00	1.00	1.00	1.00	1.00	1.00	1.00
25-29.9	0.81 (0.74, 0.89)	0.86 (0.81, 0.92)	0.98 (0.92, 1.04)	0.81 (0.74, 0.89)	0.75 (0.69, 0.82)	0.83 (0.83, 0.84)	0.77 (0.68, 0.88)	0.31 (0.16, 0.60)
30-34.9	0.86 (0.74, 0.99)	0.84 (0.76, 0.92)	1.04 (0.95, 1.13)	0.74 (0.65, 0.84)	0.79 (0.71, 0.89)	0.79 (0.79, 0.80)	0.64 (0.54, 0.75)	0.29 (0.14, 0.60)
35-39.9	0.70 (0.55, 0.88)[1]	0.87 (0.75, 1.01)	1.20 (1.06, 1.37)	0.73 (0.61, 0.88)	0.77 (0.65, 0.90)	0.74 (0.73, 0.75)	0.62 (0.50, 0.77)	0.68 (0.33, 1.43)
40+		0.97 (0.80, 1.19)	1.10 (0.87, 1.40)	0.75 (0.59, 0.95)	0.65 (0.53, 0.80)	0.67 (0.66, 0.68)	0.65 (0.50, 0.85)	0.28 (0.08, 0.95)
**MPTB**								
<18.5	0.93 (0.66, 1.30)	1.38 (1.25, 1.52)	1.21 (1.04, 1.41)	1.40 (1.12, 1.76)	1.66 (1.32, 2.09)	1.27 (1.26, 1.29)	1.42 (1.06, 1.90)	1.90 (0.53, 6.80)
18.5-24.9	1.00	1.00	1.00	1.00	1.00	1.00	1.00	1.00
25-29.9	0.75 (0.59, 0.96)	1.14 (1.08, 1.19)	1.22 (1.14, 1.31)	1.08 (0.97, 1.20)	1.02 (0.93, 1.13)	1.14 (1.13, 1.15)	1.01 (0.89, 1.13)	0.59 (0.34, 1.04)
30-34.9	1.01 (0.71, 1.44)	1.27 (1.19, 1.36)	1.59 (1.46, 1.73)	1.31 (1.15, 1.48)	1.31 (1.18, 1.46)	1.36 (1.35, 1.37)	1.08 (0.95, 1.24)	0.46 (0.24, 0.87)
35-39.9	1.28 (0.80, 2.05)[1]	1.42 (1.28, 1.57)	1.79 (1.57, 2.04)	1.34 (1.13, 1.59)	1.60 (1.40, 1.83)	1.59 (1.57, 1.60)	1.17 (0.99, 1.38)	0.26 (0.09, 0.75)
40+		1.68 (1.47, 1.92)	1.59 (1.25, 2.02)	1.55 (1.25, 1.91)	1.50 (1.28, 1.77)	1.97 (1.95, 1.98)	1.27 (1.04, 1.55)	0.76 (0.32, 1.81)

1. BMI ≥35 kg/m^2 as too few individuals with BMI>40 in this dataset

Supplementary Table S25: Adjusted odds ratios (95% CI) for any preterm, spontaneous preterm and medically indicated preterm birth by BMI category excluding multiple births – nulliparous women

BMI category	CPP, USA	Danish linked data	Norwegian birth registry	CPRD, UK	South Australian BEBOLD	US vital statistics	SAIL Databank, UK	Bradford, UK
**Any PTB**								
<18.5	1.22 (1.06, 1.40)	1.20 (1.12, 1.28)	1.23 (1.12, 1.34)	1.21 (1.06, 1.38)	1.33 (1.13, 1.56)	1.21 (1.20, 1.22)	1.32 (1.02, 1.70)	3.30 (1.33, 8.20)
18.5-24.9	1.00	1.00	1.00	1.00	1.00	1.00	1.00	1.00
25-29.9	1.00 (0.85, 1.19)	1.04 (1.00, 1.08)	1.15 (1.10, 1.21)	1.09 (1.01, 1.17)	1.02 (0.94, 1.11)	1.11 (1.11, 1.12)	1.01 (0.91, 1.12)	0.74 (0.45, 1.21)
30-34.9	0.77 (0.54, 1.09)	1.14 (1.08, 1.20)	1.32 (1.23, 1.41)	1.21 (1.10, 1.33)	1.21 (1.09, 1.34)	1.33 (1.32, 1.33)	1.06 (0.93, 1.21)	0.65 (0.38, 1.14)
35-39.9	0.71 (0.36, 1.39)[1]	1.21 (1.11, 1.31)	1.41 (1.26, 1.57)	1.28 (1.10, 1.48)	1.39 (1.21, 1.59)	1.51 (1.50, 1.52)	1.30 (1.10, 1.54)	0.78 (0.38, 1.58)
40+		1.15 (1.02, 1.31)	1.28 (1.05, 1.57)	1.20 (0.98, 1.48)	1.15 (0.96, 1.38)	1.74 (1.73, 1.76)	1.12 (0.89, 1.41)	1.38 (0.63, 2.99)
**SPTB**								
<18.5	1.24 (1.07, 1.44)	1.23 (1.12, 1.34)	1.30 (1.17, 1.44)	1.17 (1.00, 1.38)	1.45 (1.18, 1.79)	1.26 (1.25, 1.28)	1.41 (1.01, 1.95)	4.12 (1.54, 11.05)
18.5-24.9	1.00	1.00	1.00	1.00	1.00	1.00	1.00	1.00
25-29.9	0.97 (0.82, 1.16)	0.93 (0.88, 0.98)	1.04 (0.97, 1.10)	1.00 (0.91, 1.09)	0.91 (0.82, 1.01)	0.98 (0.98, 0.99)	0.96 (0.83, 1.11)	0.65 (0.35, 1.19)
30-34.9	0.64 (0.43, 0.95)	0.93 (0.86, 1.01)	1.09 (0.99, 1.19)	0.91 (0.80, 1.04)	1.04 (0.90, 1.19)	1.06 (1.05, 1.07)	0.88 (0.73, 1.06)	0.52 (0.26, 1.06)
35-39.9	0.72 (0.36, 1.46)[1]	0.85 (0.74, 0.97)	1.05 (0.90, 1.22)	0.92 (0.75, 1.13)	0.96 (0.78, 1.18)	1.09 (1.08, 1.11)	1.00 (0.78, 1.28)	0.51 (0.19, 1.38)
40+		0.80 (0.65, 0.98)	1.09 (0.84, 1.42)	0.96 (0.73, 1.27)	0.92 (0.71, 1.20)	1.12 (1.10, 1.13)	0.71 (0.49, 1.03	1.09 (0.40, 2.97)
**MPTB**								
<18.5	0.97 (0.54, 1.74)	1.16 (1.05, 1.28)	1.09 (0.93, 1.28)	1.29 (1.04, 1.60)	1.17 (0.90, 1.51)	1.13 (1.11, 1.15)	1.21 (0.83, 1.78)	1.26 (0.16, 10.18)
18.5-24.9	1.00	1.00	1.00	1.00	1.00	1.00	1.00	1.00
25-29.9	1.13 (0.63, 2.04)	1.16 (1.10, 1.22)	1.37 (1.27, 1.48)	1.26 (1.13, 1.42)	1.17 (1.05, 1.32)	1.27 (1.26, 1.28)	1.05 (0.90, 1.23)	0.95 (0.42, 2.14)
30-34.9	2.37 (1.07, 5.27)	1.38 (1.28, 1.48)	1.77 (1.60, 1.96)	1.76 (1.53, 2.03)	1.45 (1.26, 1.68)	1.66 (1.64, 1.67)	1.28 (1.07, 1.53)	0.97 (0.40, 2.33)
35-39.9	1.02 (0.14, 7.53)[1]	1.61 (1.44, 1.80)	2.12 (1.82, 2.48)	1.99 (1.63, 2.42)	1.97 (1.65, 2.35)	2.03 (2.00, 2.05)	1.67 (1.34, 2.08)	1.38 (0.50, 3.82)
40+		1.56 (1.33, 1.83)	1.66 (1.23, 2.24)	1.65 (1.23, 2.21)	1.46 (1.14, 1.87)	2.51 (2.48, 2.54)	1.62 (1.22, 2.15)	1.90 (0.59, 6.19)

1. BMI ≥35 kg/m^2 as too few individuals with BMI>40 in this dataset

Supplementary Table S26: Adjusted odds ratios (95% CI) for any preterm, spontaneous preterm and medically indicated preterm birth by BMI category excluding multiple births – parous women

**Any PTB**								
BMI category	CPP, USA	Danish linked data	Norwegian birth registry	CPRD, UK	South Australian BEBOLD	US vital statistics	SAIL Databank, UK	Bradford, UK
<18.5	1.23 (1.11, 1.37)	1.54 (1.43, 1.66)	1.46 (1.33, 1.61)	1.56 (1.35, 1.79)	1.80 (1.55, 2.09)	1.47 (1.46, 1.49)	1.61 (1.31, 1.97)	2.78 (1.21, 6.38)
18.5-24.9	1.00	1.00	1.00	1.00	1.00	1.00	1.00	1.00
25-29.9	0.79 (0.72, 0.86)	1.03 (0.99, 1.07)	1.08 (1.03, 1.13)	0.92 (0.85, 0.99)	0.88 (0.82, 0.94)	0.98 (0.98, 0.99)	0.87 (0.80, 0.96)	0.41 (0.27, 0.65)
30-34.9	0.81 (0.70, 0.92)	1.08 (1.02, 1.15)	1.29 (1.21, 1.38)	0.98 (0.89, 1.08)	1.00 (0.91, 1.09)	1.07 (1.07, 1.08)	0.84 (0.75, 0.94)	0.34 (0.20, 0.57)
35-39.9	0.72 (0.59, 0.89)[1]	1.19 (1.09, 1.30)	1.47 (1.34, 1.63)	0.96 (0.84, 1.09)	1.13 (1.01, 1.26)	1.16 (1.15, 1.16)	0.88 (0.76, 1.01)	0.45 (0.24, 0.83)
40+		1.41 (1.25, 1.59)	1.39 (1.16, 1.66)	1.06 (0.90, 1.26)	1.00 (0.87, 1.15)	1.31 (1.30, 1.32)	1.00 (0.85, 1.17)	0.47 (0.22, 1.02)
**SPTB**								
<18.5	1.28 (1.14, 1.43)	1.69 (1.52, 1.88)	1.62 (1.44, 1.82)	1.61 (1.35, 1.92)	1.85 (1.53, 2.23)	1.61 (1.59, 1.63)	1.80 (1.39, 2.33)	3.12 (1.17, 8.37)
18.5-24.9	1.00	1.00	1.00	1.00	1.00	1.00	1.00	1.00
25-29.9	0.79 (0.72, 0.86)	0.86 (0.81, 0.92)	0.97 (0.91, 1.04)	0.81 (0.73, 0.90)	0.73 (0.66, 0.81)	0.83 (0.83, 0.84)	0.77 (0.68, 0.88)	0.26 (0.13, 0.53)
30-34.9	0.80 (0.69, 0.93)	0.83 (0.75, 0.91)	1.05 (0.96, 1.14)	0.73 (0.63, 0.83)	0.73 (0.65, 0.83)	0.79 (0.79, 0.80)	0.61 (0.51, 0.72)	0.26 (0.12, 0.58)
35-39.9	0.67 (0.53, 0.84)[1]	0.89 (0.76, 1.04)	1.21 (1.05, 1.39)	0.72 (0.60, 0.86)	0.75 (0.64, 0.89)	0.74 (0.73, 0.74)	0.60 (0.47, 0.75)	0.68 (0.33, 1.44)
40+		1.00 (0.81, 1.23)	1.17 (0.92, 1.50)	0.74 (0.57, 0.95)	0.63 (0.51, 0.79)	0.66 (0.65, 0.67)	0.67 (0.51, 0.87)	0.35 (0.10, 1.17)
**MPTB**								
<18.5	0.96 (0.68, 1.34)	1.43 (1.29, 1.58)	1.22 (1.04, 1.44)	1.46 (1.16, 1.85)	1.73 (1.36, 2.18)	1.28 (1.27, 1.30)	1.39 (1.02, 1.88)	1.80 (0.50, 6.44)
18.5-24.9	1.00	1.00	1.00	1.00	1.00	1.00	1.00	1.00
25-29.9	0.71 (0.55, 0.92)	1.15 (1.09, 1.21)	1.23 (1.15, 1.32)	1.08 (0.97, 1.21)	1.07 (0.97, 1.19)	1.13 (1.12, 1.14)	0.98 (0.87, 1.11)	0.61 (0.34, 1.07)
30-34.9	0.94 (0.65, 1.35)	1.28 (1.19, 1.37)	1.65 (1.51, 1.80)	1.38 (1.21, 1.57)	1.35 (1.20, 1.52)	1.35 (1.34, 1.36)	1.08 (0.93, 1.24)	0.46 (0.23, 0.89)
35-39.9	1.07 (0.66, 1.74)[1]	1.41 (1.27, 1.57)	1.86 (1.63, 2.13)	1.33 (1.11, 1.59)	1.63 (1.41, 1.88)	1.56 (1.55, 1.58)	1.15 (0.97, 1.37)	0.26 (0.09, 0.76)
40+		1.71 (1.48, 1.97)	1.70 (1.33, 2.18)	1.57 (1.26, 1.95)	1.51 (1.26, 1.80)	1.93 (1.91, 1.95)	1.33 (1.08, 1.62)	0.64 (0.24, 1.71)

1. BMI ≥35 kg/m^2 as too few individuals with BMI>40 in this dataset

Supplementary Figure S2: Meta-analysis of association of pre-pregnancy BMI with any PTB, SPTB and MPTB including and omitting CPP – nulliparous women

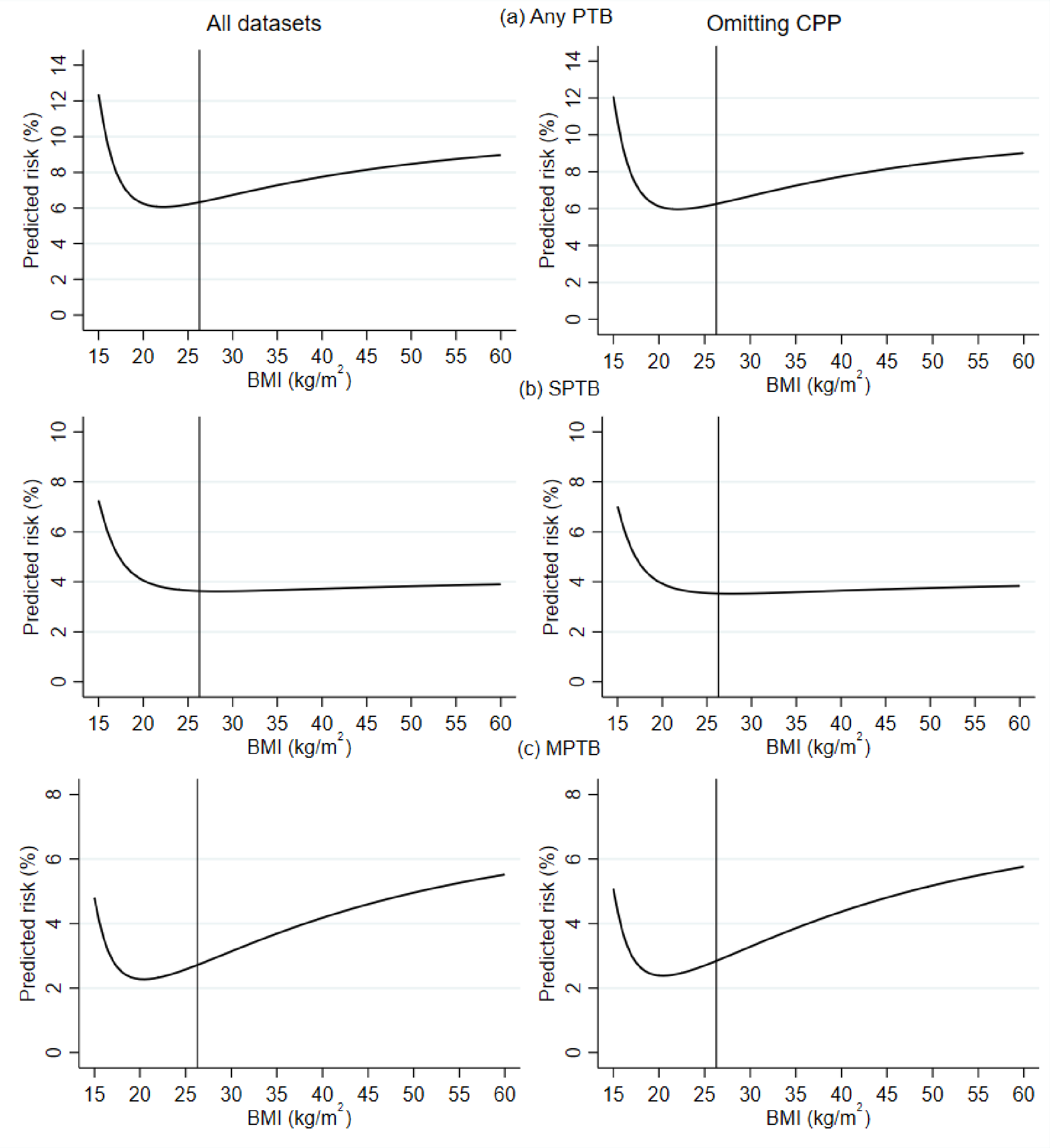

Supplementary Figure S3: Meta-analysis of association of pre-pregnancy BMI with any PTB, SPTB and MPTB including and omitting CPP – parous women

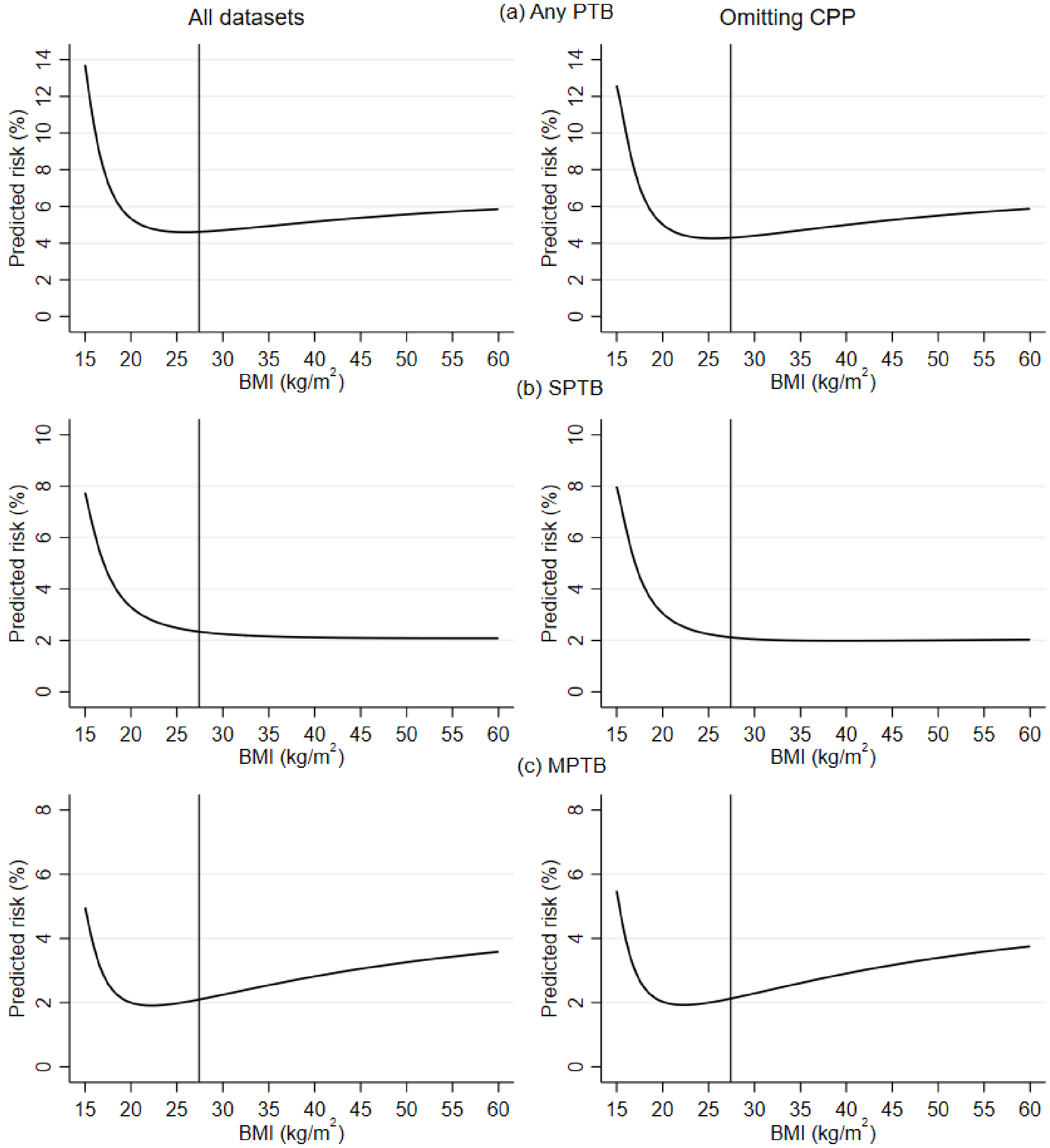

Supplement: Supplementary file 1 — Additional file 1: Supplementary text A.1. Further details about the datasets and availability of confounders. Supplementary text A.2. Further details of statistical methods. Supplementary Figure S1. Summary of missing data. Supplementary Table S1-S8. For each dataset- characteristics of the whole sample, complete cases and excluded cases. Supplementary Tables S9-S10. Univariate risk of any PTB, SPTB and MPTB. Supplementary Tables S11-S12. Deviance from different fractional polynomials. Supplementary Tables S13-S14. Meta-analysis results. Supplementary Tables S15-S16. Adjusted odds ratios of any PTB, SPTB and MPTB by BMI category. Supplementary Tables S17-S18. Adjusted odds ratios for any very PTB, spontaneous very PTB and medically indicated very PTB by BMI category. Supplementary Tables S19-S20. Adjusted odds ratios for any PTB, SPTB and MPTB – inverse probability weighted. Supplementary Tables S21-S22. Adjusted odds ratios for any PTB, SPTB and MPTB excluding stillbirths. Supplementary Tables S23-S24. Adjusted odds ratios for any PTB, SPTB and MPTB excluding post term births. Supplementary Tables S25-S26. Adjusted odds ratios for any PTB, SPTB and MPTB excluding multiple births. Supplementary Figures S1-S2. Meta-analysis including and omitting CPP. [file 12916_2023_3230_MOESM1_ESM.pdf]
